# The Contribution of Phospholipase C in Vomiting in the Least Shrew (Cryptotis Parva) Model of Emesis

**DOI:** 10.3389/fphar.2021.736842

**Published:** 2021-09-10

**Authors:** Weixia Zhong, Nissar A. Darmani

**Affiliations:** Department of Basic Medical Sciences, College of Osteopathic Medicine of the Pacific, Western University of Health Sciences, Pomona, CA, United States

**Keywords:** PLC, m-3M3FBS, U73122, emesis, dorsal vagal complex, least shrew

## Abstract

Gq and Gβγ protein-dependent phospholipase C (PLC) activation is extensively involved in G protein-coupled receptor (GPCR)-mediated signaling pathways which are implicated in a wide range of physiological and pathological events. Stimulation of several GPCRs, such as substance P neurokinin 1-, dopamine D_2/3_-, histamine H_1_- and mu-opioid receptors, can lead to vomiting. The aim of this study was to investigate the role of PLC in vomiting through assessment of the emetic potential of a PLC activator (m-3M3FBS), and the antiemetic efficacy of a PLC inhibitor (U73122), in the least shrew model of vomiting. We find that a 50 mg/kg (i.p.) dose of m-3M3FBS induces vomiting in ∼90% of tested least shrews, which was accompanied by significant increases in c-Fos expression and ERK1/2 phosphorylation in the shrew brainstem dorsal vagal complex, indicating activation of brainstem emetic nuclei in m-3M3FBS-evoked emesis. The m-3M3FBS-evoked vomiting was reduced by pretreatment with diverse antiemetics including the antagonists/inhibitors of: PLC (U73122), L-type Ca^2+^ channel (nifedipine), IP_3_R (2-APB), RyR receptor (dantrolene), ERK1/2 (U0126), PKC (GF109203X), the serotoninergic type 3 receptor (palonosetron), and neurokinin 1 receptor (netupitant). In addition, the PLC inhibitor U73122 displayed broad-spectrum antiemetic effects against diverse emetogens, including the selective agonists of serotonin type 3 (2-Methyl-5-HT)-, neurokinin 1 receptor (GR73632), dopamine D_2/3_ (quinpirole)-, and muscarinic M_1_ (McN-A-343) receptors, the L-type Ca^2+^ channel (FPL64176), and the sarco/endoplasmic reticulum Ca^2+^-ATPase inhibitor thapsigargin. In sum, PLC activation contributes to emesis, whereas PLC inhibition suppresses vomiting evoked by diverse emetogens.

## Highlights

The PLC activator m-3M3FBS is proemetic in the least shrew.
m-3M3FBS evokes c-Fos expression and ERK1/2 phosphorylation in the brainstem emetic nuclei.
ERK1/2 and PKC inhibitors, Ca^2+^ channel modulators, and serotonin 5-HT_3_/neurokinin NK_1_ receptor antagonists reduce m-3M3FBS-induced vomiting.
The PLC inhibitor U73122 exerts antiemetic effects against the vomiting-evoked by various emetogens including m-3M3FBS.

## Introduction

The emetic nuclei involved in vomiting include the dorsal vagal complex (DVC) [containing the area postrema (AP), nucleus tractus solitarius (NTS) and dorsal motor nucleus of the vagus (DMNV)] in the brainstem, as well peripheral loci such as neurons of the enteric nervous system and enterochromaffin cells which are embedded in the lining of the gastrointestinal tract, as well as vagal afferents which carry input from the gastrointestinal tract to the brainstem DVC (Darmani and Ray, 2009; Babic and Browning, 2014). It is well recognized that the numerous receptors involved in vomiting are located both in the periphery such as the gastrointestinal tract as well as in the brainstem DVC emetic nuclei (Navari, 2014; Wickham, 2020). Receptors that mediate vomiting include opioid mu and kappa, dopamine D_2_ and D_3_, substance P neurokinin 1 (NK_1_), serotonin type 3 (5-HT_3_), histamine H_1_, muscarinic M_1_ (Beleslin and Nedelkovski, 1988), and neuropeptide Y_2_ receptors, just to name a few (MacDougall and Sharma, 2020). All the above discussed emetic receptors except 5-HT_3_ receptors, belong to the G protein-coupled receptor family (GPCRs) which are involved in a myriad of physiological functions (Ilyaskina et al., 2018). GPCRs can couple to a family of Gα-protein subclasses (G_i/o_, G_q/11_, G_s_, and G_12/13_) as well as the G_βγ_ subunits (Ilyaskina et al., 2018). In brief, in the inactive state the G-protein exists as an αβγ trimer complex and following agonist activation a conformational change in the GPCR occurs which leads to its association with an α subunit and the dissociation of the G_βγ_ subunit (Jo and Jung, 2016). Among the α-subunits, G_q/11_ protein activates phospholipase C (PLC) to generate inositol 1,4,5-trisphosphate (IP_3_) and diacylglycerol (DAG). Cytosolic IP_3_ subsequently increases intracellular Ca^2+^ concentration via the IP_3_ receptor (IP_3_R)-mediated release of Ca^2+^ from the endoplasmic reticulum calcium stores into the cytoplasm which then triggers protein kinase C (PKC) phosphorylation/activation, as well as further activation of multiple protein kinases including extracellular signal-regulated kinase1/2 (ERK1/2) (Goldsmith and Dhanasekaran, 2007). The dimer G_βγ_ is also able to activate ERK1/2 through PLC-dependent or phosphoinositide 3-kinase-dependent pathways (Zhao et al., 2016). Several studies indicate that IP_3_ production is involved in the induction of vomiting (Hagbom et al., 2011; Kawakami and Xiao, 2013; Hille et al., 2015). Thus, PLC activation following activation G_q/11_ and the G_βγ_ proteins represents an important factor in GPCRs signaling pathways in diverse physiological functions and pathological conditions (Hagbom et al., 2011; Kawakami and Xiao, 2013; Hille et al., 2015). In addition, activation of the opioid mu (Smart et al., 1997; Rubovitch et al., 2003)-, opioid kappa (Murthy and Makhlouf, 1996; Joshi et al., 1999), dopamine D_2_ (Fregeau et al., 2013; Jijon-Lorenzo et al., 2018)-, dopamine D_3_ (Pedrosa et al., 2004)-, substance P neurokinin NK_1_ (NK_1_) (Javid et al., 2019)-, histamine H_1_ (Parsons and Ganellin, 2006)-, muscarinic M_1_ (Michal et al., 2015; Ilyaskina et al., 2018; Maeda et al., 2019)-, and neuropeptide Y_2_-receptors (Domingues et al., 2018; Tan et al., 2018; Ziffert et al., 2020), lead to PLC1-coupled signaling events.

In the present study we sought to investigate the emetic potential of the widely used pharmacological tool, the PLC activator m-3M3FBS (Krjukova et al., 2004; Kim et al., 2012), and the antiemetic efficacy of the PLC inhibitor U73122 (Kim et al., 2012). Thus, we initially investigated whether intraperitoneal (i.p.) administration of varying doses of m-3M3FBS can evoke vomiting in the least shrew animal model of vomiting. Secondly, we examined the central and peripheral involvement of emetic loci underlying a maximally effective emetic-dose of m-3M3FBS (50 mg/kg, i. p.) by means of c-Fos and phospho-ERK1/2 immunostaining to indicate whether m-3M3FBS activates the brainstem dorsal vagal complex and/or the jejunum. Thereafter, we determined the receptor/signaling mechanisms by which m-3M3FBS evokes vomiting via the use of antiemetics including the: 1) PLC inhibitor, U73122; 2) ERK1/2 inhibitor, U0126 (Hotokezaka et al., 2002); 3) L-type Ca^2+^ channel (LTCC) antagonist, nifedipine (Triggle, 2007); 4) IP_3_R inhibitor 2-APB (Ng et al., 2007); 5) ryanodine receptor (RyR) inhibitor, dantrolene (Ng et al., 2007); 6) PKC inhibitor, GF109203X (Hauss et al., 1993); 7) 5-HT_3_R antagonist, palonosetron (Navari, 2013; Rojas et al., 2014); and 8) substance P neurokinin NK_1_ receptor (NK_1_R) antagonist, netupitant (Navari, 2013; Rojas et al., 2014). Although the PLC inhibitor U73122 is yet to be investigated, our previously published studies demonstrate that pretreatment with the above discussed drugs exert antiemetic effects against corresponding emetogens (Darmani et al., 2014; Darmani et al., 2015; Zhong et al., 2018; Zhong et al., 2019).

Specific emetogens such as selective agonists of 5-HT_3_ (2-Methyl-5-HT)-, NK_1_ (GR73632)-, dopamine D_2_ (quinpirole)-, and muscarinic M_1_ (McN-A-343)-receptors, as well as Ca^2+^ channel regulators including the LTCC agonist FPL6417, and the sarco/endoplasmic reticulum Ca^2+^-ATPase (SERCA) inhibitor thapsigargin, evoke pronounced vomiting in the least shrews (Darmani et al., 2014; Zhong et al., 2016). Thus, we further investigated whether pharmacological inhibition of the PLC with U73122 displays antiemetic effects against some or all of the above discussed emetogens. Overall, our results suggest the importance PLC in diverse causes of emesis.

## Materials and Methods

### Animals

A colony of adult least shrews from the Western University of Health Sciences Animal Facilities were housed in groups of 5–10 on a 14:10 light:dark cycle, and were fed and watered ad libitum. The experimental shrews were 45–60 days old and each weighed between 4 and 6 g. Animal experiments were conducted in accordance with the principles and procedures of the National Institutes of Health Guide for the Care and Use of Laboratory Animals. All protocols were approved by the Institutional Animal Care and Use Committee of Western University of Health Sciences (Protocol number R20IACUC018). All efforts were made to minimize animals suffering and to reduce the number of animals used in the experiments.

### Chemicals

The following drugs were used: m-3M3FBS, U73122, U0126, GF109203X, FPL64176, 2-methyl-serotonin maleate salt (2-Methyl-5-HT), and GR73632 were purchased from Tocris (Minneapolis, MN); McN-A-343, quinpirole HCl and nifedipine from Sigma Sigma/RBI (St. Louis, MO); thapsigargin, dantrolene and 2-APB from Santa Cruz Biotechnology (Dallas, TX). Palonosetron and netupitant were kindly provided by Helsinn Health Care (Lugano, Switzerland). m-3M3FBS, U73122, nifedipine, U0126, netupitant were dissolved in a mixture of ethanol/Tween 80/saline at a volume ratio of 1:1:18. Dantrolene, 2-APB, GF109203X and FPL64176 were dissolved in 25% DMSO in water. Thapsigargin was dissolved in 10% DMSO in distilled water. Other drugs were dissolved in distilled water. All drugs were administered at a volume of 0.1 ml/10 g of body weight.

### Behavioral Emesis Studies

On the day of experimentation shrews were brought from the animal facility, separated into individual cages, and allowed to adapt for at least 2 hours (h). Daily food was withheld 2 h prior to the start of the experiment, but shrews were given 4 mealworms each prior to emetogen injection to aid in identifying wet vomits as described previously (Darmani, 1998). For systemic dose-response emesis studies, different groups of shrews were injected with varying doses of m-3M3FBS (0, 10, 20, and 50 mg/kg, i. p., *n* = 8 shrews per group). Each shrew was immediately placed in the observation cage and the frequency of emesis was recorded for the next 2 h m-3M3FBS at 50 mg/kg dose caused vomiting with maximal frequency in 87.5% of tested shrews. Thus, this dose was used for subsequent studies.

To evaluate drug interaction studies, different groups of shrews were pretreated for 30 min with an injection of either corresponding vehicle [(i.p.) or subcutaneously (s.c.)], or varying doses of the following antiemetic antagonists/inhibitors: 1) PLC inhibitor U73122 (2.5 and 10 mg/kg, i. p., *n* = 8 per group); 2) ERK1/2 inhibitor U1026 (1 and 5 mg/kg, i. p., *n* = 8 per group); 3) IP_3_R antagonist 2-APB (0.5, 2.5 and 10 mg/kg, i. p., *n* = 8 per group); 4) RyR antagonist dantrolene (0.5, 2.5 and 10 mg/kg, i. p., *n* = 8 per group); 5) LTCC blocker nifedipine (0.5 and 1 mg/kg, s. c., *n* = 10 per group); 6) PKC inhibitor GF109203X (0.1, 1 and 10 mg/kg, i. p., *n* = 8); 7) 5-HT_3_R antagonist palonosetron (0.1 and 0.5 mg/kg, s. c., *n* = 8 per group); 8) NK_1_R antagonist netupitant (1, and 5 mg/kg, i. p., *n* = 8 per group). After 30 min pretreatment, each shrew was challenged with a single dose of m-3M3FBS at 50 mg/kg (i.p.) and emesis behaviors were recorded for the following 2 h. The selection of dosage levels of the above tested drugs were based up on our previous antiemetic studies (Zhong et al., 2019).

To determine the broad-spectrum dose-response antiemetic potential of the PLC inhibitor U73122, varying doses were injected (i.p.) into different groups of shrews at 0 min. Thirty minutes later, shrews were challenged for vomiting with a fully efficacious dose of one of the following emetogens (Darmani et al., 2014; Zhong et al., 2016; 2014): 1) the selective serotonin 5-HT_3_R agonist 2-Methyl-5-HT (5 mg/kg, i. p., *n* = 8 per group), 2) the selective substance P neurokinin NK_1_R agonist GR73632 (5 mg/kg, i. p., *n* = 6 per group), 3) the dopamine D_2/3_ preferring receptor agonist quinpirole (2 mg/kg, i. p., *n* = 5 per group), 4) the selective muscarinic M_1_R agonist McN-A-343 (2 mg/kg, i. p., *n* = 6 per group), 5) the LTCC agonist FPL64176 (10 mg/kg, i. p., *n* = 6 per group), 6) the SERCA inhibitor thapsigargin (0.5 mg/kg, i. p., *n* = 6 per group). The vomiting behavior (number of animals vomiting within groups and frequency of vomits) were then observed for 30 min.

In the forementioned emesis behavioral experiments, the observer was blinded to administration conditions. In all experiments each tested shrew was used once and then euthanized with isoflurane following the termination of each experiment.

### c-Fos Immunostaining and Image Analysis

Immunohistochemistry of the least shrew brainstem and jejunal sections was conducted as previously reported (Zhong et al., 2019; Zhong et al., 2021). The jejunal segment of the least shrew small intestine was dissected as described by Ray et al. (2009). Following m-3M3FBS (50 mg/kg, i. p.) injection, vomiting shrews were subjected to c-Fos staining (*n* = 3–4 shrews/group). Thus, 90 min after the first emesis occurred, shrews were anesthetized with isoflurane and perfused with ice cold 4% paraformaldehyde in pH 7.4, 0.1 M phosphate-buffered saline (PBS) for 10 min. Brainstems and jejunum were removed and cryoprotected with 30% sucrose in 0.01 M PBS overnight. The OCT-embedded brainstem block and jejunum were cut on a freezing microtome (Leica, Bannockburn, IL, United States) into 20-μm and 25-μm sections respectively and stored in PBS with 0.03% sodium azide. Immunolabeling using rabbit anti-c-Fos polyclonal antibody (1:5,000, ab190289, Abcam) were performed. Alexa Fluor 594 donkey anti-rabbit IgG (1:500, Invitrogen) was used as secondary antibody. Nuclei of cells were stained with DAPI. Images of the brainstem sections containing the dorsal vagal complex (AP/NTS/DMNV) and jejunal sections were taken by a confocal microscope (Zeiss LMS 880) with Zen software using ×20 objective. Cytoarchitectonic differences in the AP, NTS, and DMNV of the least shrew brainstem have been described in our previous report (Ray et al., 2009).

A Fos-expressing cell nucleus was only counted as positive if it retained its ovoid shape after high-pass filtering to eliminate variations in background as well as potential false positive and was fully within the defined region of interest. The numbers of Fos-positive nuclei of each region (AP/NTS/DMNV/jejunum) were counted by an observer blind to the animal’s treatment condition. For each region, the same number of sections were counted per animal: 3 brainstem sections at 90-μm intervals each through AP/NTS/DMNV and 3 jejunal sections. The mean value of each region per section, from an individual animal was used in statistical analysis.

### Phospho-ERK1/2 Immunohistochemistry

Adult least shrews were administered m-3M3FBS (50 mg/kg, i. p.) or vehicle (*n* = 3 animals per group) and rapidly anesthetized with isoflurane and subjected to perfusion at 15 min post-treatment to evaluate phospho-ERK1/2 alteration. Brain sections (20 μm) observed under a light microscope and those containing the brainstem dorsal vagal complex (DVC) were subjected to immunostaining as described in our previous publication (Zhong et al., 2019). Immunostaining using rabbit anti-phospho-ERK1/2 (Thr202/Thr204) (1:500, 4,370, Cell Signaling) antibody was followed by Alexa Fluor 594 donkey anti-rabbit IgG (1:500, Invitrogen) secondary antibody incubation. After washing with PBS 4 times, sections were mounted with anti-fade mounting medium containing DAPI staining nuclei (Vector Laboratories). Images were acquired under a confocal microscope (Zeiss) with Zen software using ×20 and ×60 objectives. Integrated density of phospho-ERK1/2 immunoreactivity in the brainstem dorsal vagal complex of 3 sections from each animal of both groups was determined with ImageJ and the mean value per section from individual animals was used in statistical analysis.

### Statistical Analysis

The vomit frequency data were analyzed using the Kruskal-Wallis non-parametric one-way analysis of variance (ANOVA) followed by Dunnett’s post hoc test and expressed as the mean ± SEM. The percentage of animals vomiting across groups at different doses was compared using the chi-square test. Statistical significance for differences of c-Fos expression between two groups (vehicle controls vs. shrews vomiting induced by m-3M3FBS) was tested by unpaired *t*-test. *p* < 0.5 was considered statistically significant.

## Results

### m-3M3FBS Induces Emesis

**FIGURE 1:**
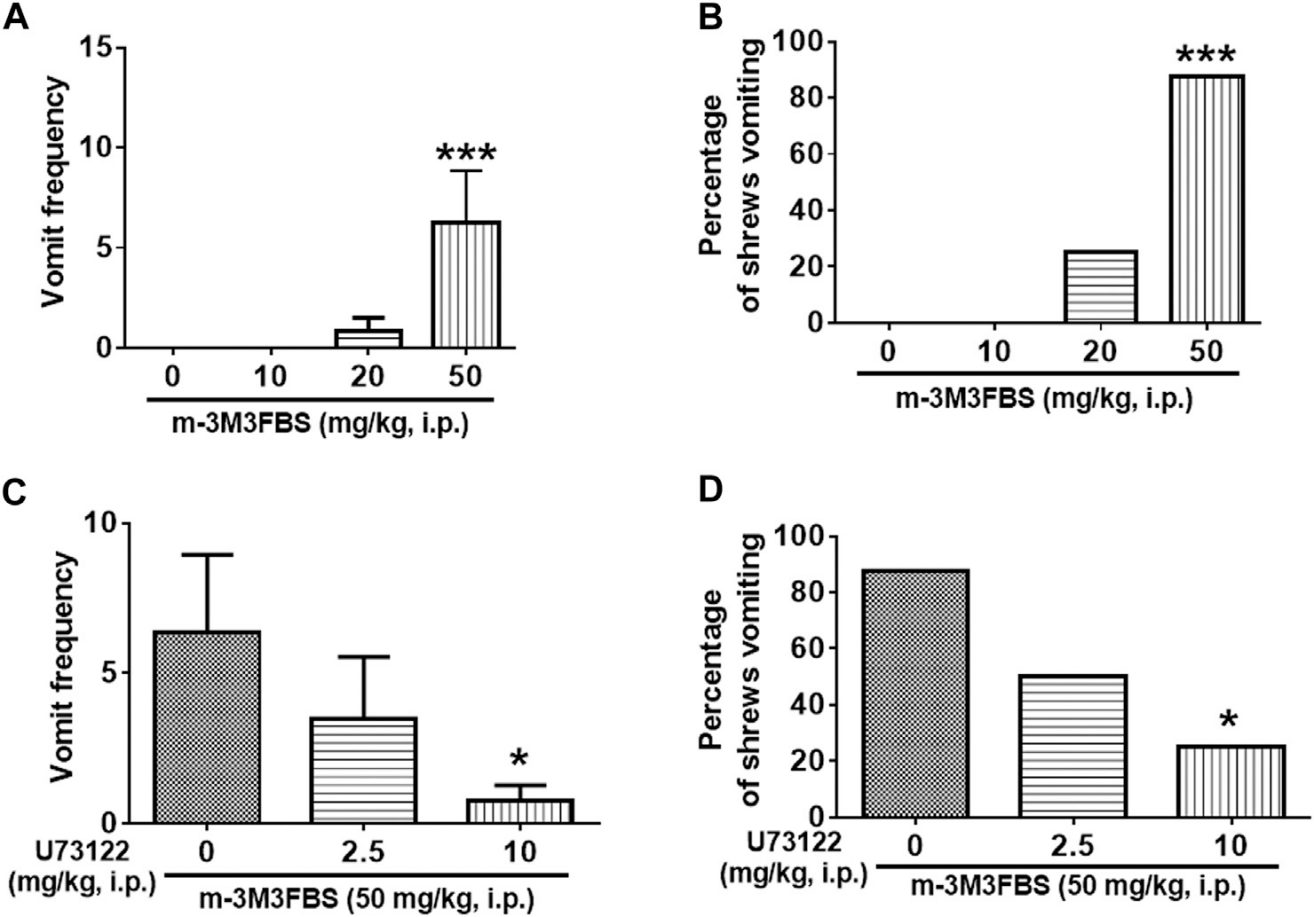
The Proemetic effects of the PLC activator m-3M3FBS and the corresponding antiemetic efficacy of the PLC inhibitor U73122 in least shrews. **(A,B)** Different groups of least shrews were given varying doses of m-3M3FBS (i.p., *n* = 8 shrews per group). Emetic parameters were recorded for the next 2 h. **(C,D)** In drug interaction studies, different groups of least shrews were given an injection (i.p.) of either the corresponding vehicle, or varying doses of U73122, 30 minutes prior to an injection of m-3M3FBS (50 mg/kg, i. p.), and were observed for the next 2 h. **(A,C)** The frequency of emesis was analyzed with Kruskal-Wallis non-parametric one-way ANOVA followed by Dunnett’s post hoc test and presented as mean ± SEM. **(B,D)** Percentage of shrews vomiting was analyzed with chi-square test and presented as mean. *p < 0.05, ***p < 0.001vs. 0 mg/kg.

The emesis data for m-3M3FBS are depicted in Figure 1. Intraperitoneal administration of m-3M3FBS increased the frequency of emesis in the least shrew in a dose-dependent manner (KW (3, 28) = 19.51, *p* = 0.0002). Dunn’s multiple comparisons post hoc test showed that m-3M3FBS significantly increased the vomit frequency at its 50 mg/kg dose (*p* = 0.0004) (Figure 1A). In addition, the chi-square test indicated that the percentage of animals exhibiting emesis in response to m-3M3FBS also increased in a dose-dependent fashion (χ^2^ (3, 28) = 20.25, *p* = 0.0002). However, only 87.5% of shrews vomited at its 50 mg/kg (*p* = 0.0004) (Figure 1B). We could not test larger doses of m-3M3FBS due to its insolubility.

As shown in Figure 1C, the PLC inhibitor U73122 attenuated the frequency of m-3M3FBS -induced vomiting [KW (2, 21) = 6.564, *p* = 0.0375], with a significant reduction (88%) seen at its 10 mg/kg dose (*p* = 0.0209). U73122 also reduced the percentage of shrews vomiting [χ^2^ (2, 21) = 6.378; *p* = 0.0412], and significant protection (62.5%) occurred at 10 mg/kg (*p* = 0.0117) (Figure 1D).

### m-3M3FBS Activates Brainstem Emetic Nuclei

**FIGURE 2:**
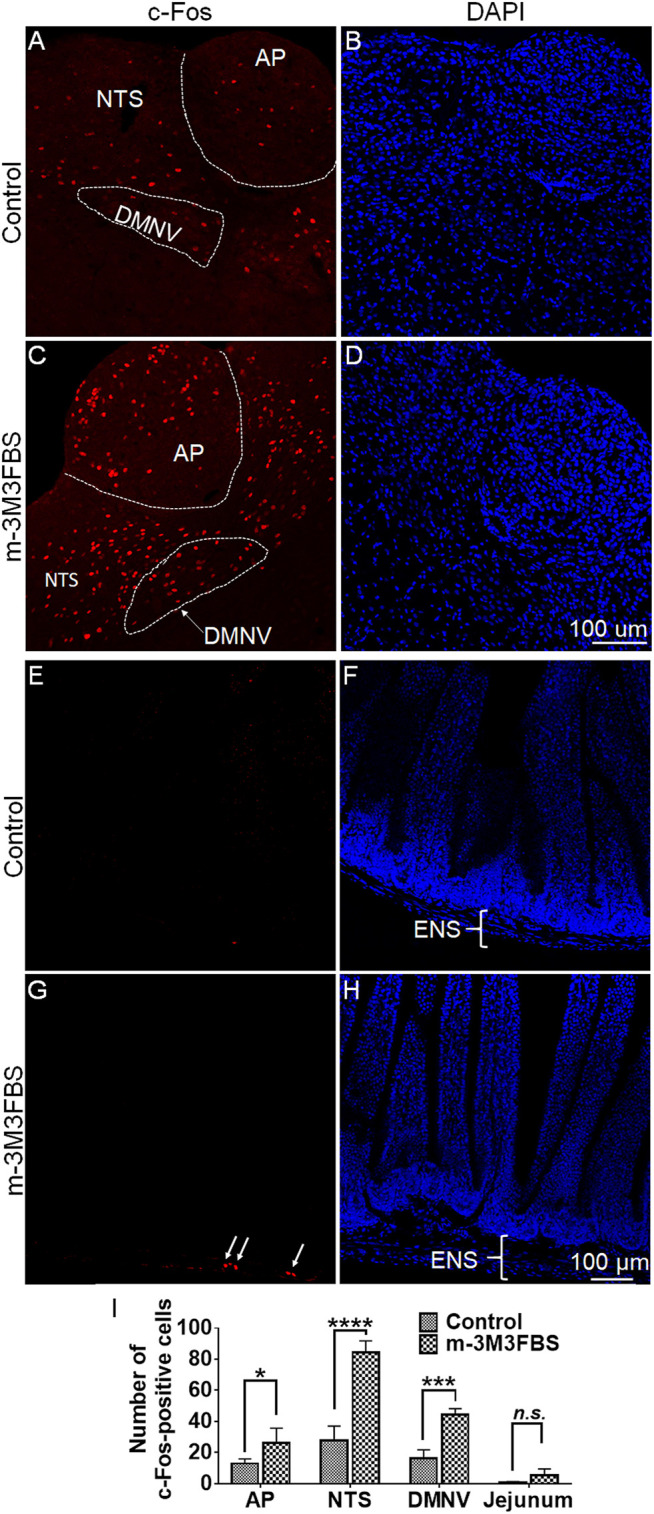
Immunohistochemical analysis of c-Fos expression following m-3M3FBS-induced emesis. Least shrews were sacrificed 90 min post vehicle treatment, or after the first vomiting occurred post systemic administration (50 mg/kg, i. p.) of m-3M3FBS (*n* = 3 shrews per group). Shrew brainstem sections (20 μm) and jejunal sections (25 μm) were stained with rabbit c-Fos antibody and Alexa Fluor 594 donkey anti-rabbit secondary antibody. **(A,C)** Representative images show that compared to the vehicle-treated control group, significant c-Fos expression evoked by m-3M3FBS was observed in the brainstem dorsal vagal complex emetic nuclei, the area postrema (AP), the nucleus tractus solitarius (NTS) and the dorsal motor nucleus of the vagus (DMNV). **(B,D)** Nuclei were stained with DAPI in blue. Scale bar, 100 μm. **(E,G)** Representative images show that compared to the vehicle-treated control group, a weak c-Fos expression following m-3M3FBS-induced vomiting was peripherally observed in the enteric nervous system (ENS) embedded in the wall of the jejunum of the small intestine. **(F,H)** Nuclei were stained with DAPI in blue. Scale bar, 100 μm. **(I)** Quantified data for the of m-3M3FBS-induced c-Fos expression in the least shrew brainstem dorsal vagal complex and jejunum. Values represent the mean number of c-Fos-positive nuclei of each region (AP/NTS/DMNV/jejunum) per section and is presented as mean ± SEM (n = 3 shrews per group). *p < 0.05, ***p < 0.001, ****p < 0.0001 vs. Control (treated with vehicle of m-3M3FBS), Unpaired *t*-test.

We conducted immunohistochemistry to determine c-Fos responsiveness following systemic administration of m-3M3FBS. Figures 2A,B show very few of c-Fos-positive cells were observed in the dorsal vagal complex (DVC) emetic nuclei in shrew brainstem sections from vehicle-treated controls. Relative to the vehicle-treated control group, a 50 mg/kg (i.p.) dose of m-3M3FBS caused a significant increase in c-Fos expression in the brainstem throughout the three DVC emetic nuclei, the AP, NTS and DMNV (Figures 2C,D). The numbers of Fos-IR positive cell nuclei in each region are delineated in Figure 2I. In vehicle-treated shrews, the average values for Fos-positive cells were 12.9 ± 1.5, 27.8 ± 4.7, and 16.6 ± 2.6 in the AP, NTS, and DMNX, respectively. Following vomiting induced by m-3M3FBS, the average numbers of c-Fos-positive cells were increased to 26.3 ± 4.7 in the AP (*p* = 0.0361 vs. Control), 84.7 ± 3.6 in NTS (*p* < 0.0001), and 44.7 ± 1.8 in DMNX (*p* = 0.0001). The c-Fos expression following m-3M3FBS-induced vomiting was also examined in the shrew jejunum (Figures 2E–H). The mean number of c-Fos expressing cells in the enteric nervous system of the jejunum was 0.5278 ± 0.3938 in the vehicle-treated shrews, and 5.222 ± 2.164 after m-3M3FBS-evoked vomiting (*p* = 0.0768, vehicle vs. m-3M3FBS) (Figure 2I).

### m-3M3FBS-Induced Emesis Involves ERK1/2 Phosphorylation

**FIGURE 3:**
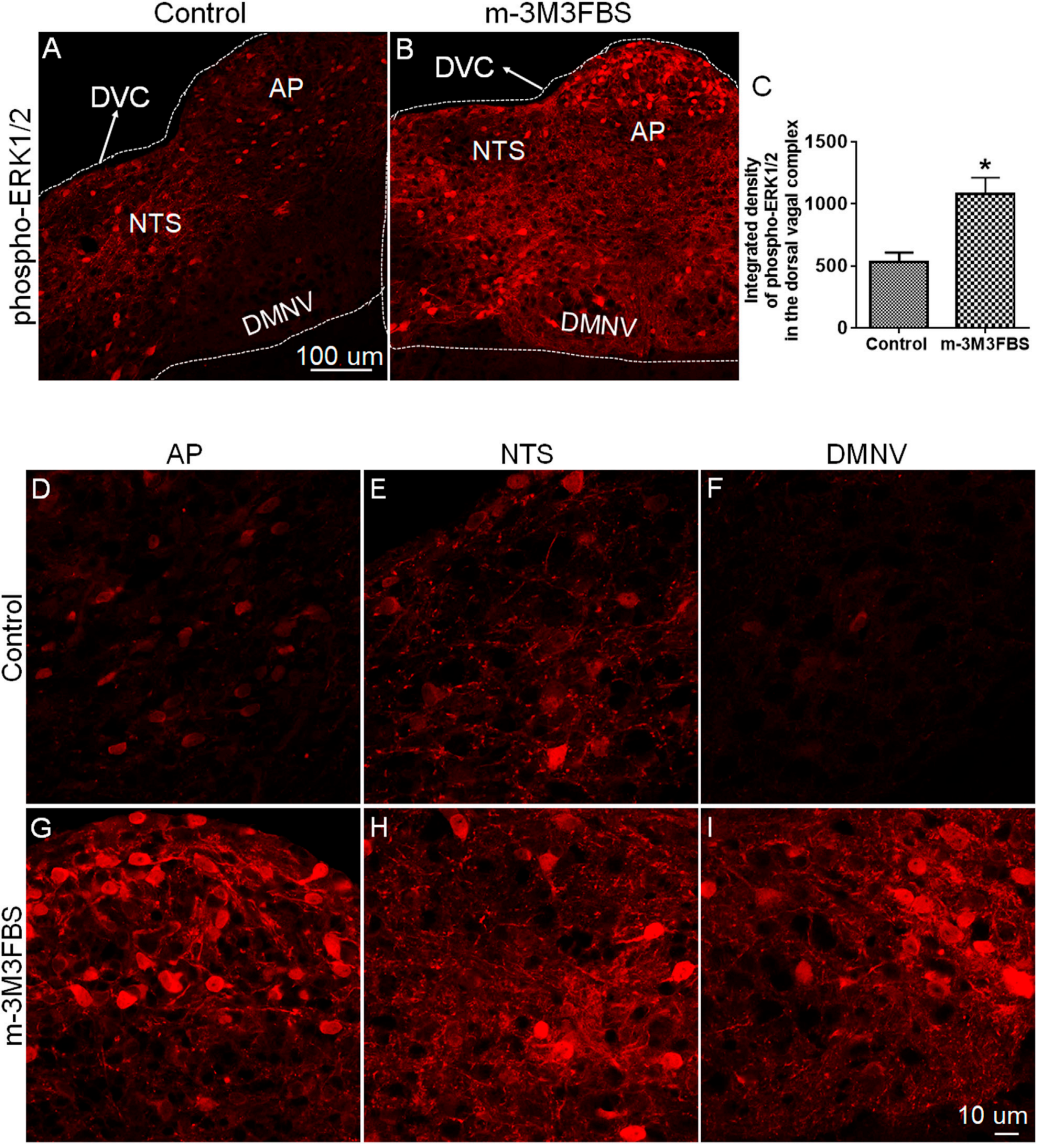
Immunohistochemical analysis of ERK1/2 phosphorylation following m-3M3FBS-induced emesis. Least shrews were sacrificed 60 min after vehicle or m-3M3FBS administration (50 mg/kg, i. p.) (*n* = 3 shrews per group). Brainstem sections (20 μm) were stained with rabbit anti-phospho-ERK1/2 antibody and Alexa Fluor 594 donkey anti-rabbit secondary antibody. **(A,B)** Representative images show that compared to the vehicle-treated control group, m-3M3FBS increased ERK1/2 phosphorylation in the brainstem dorsal vagal complex. Scale bar, 100 μm. **(C)** Statistical analysis of integrated density of ERK1/2 phosphorylation in the brainstem dorsal vagal complex. *p < 0.05 vs. Control, Unpaired *t*-test. **(D–I)** Representative magnified images show m-3M3FBS increased ERK1/2 phosphorylation in the emetic nuclei located in the dorsal vagal complex, area postrema (AP), the nucleus tractus solitarius (NTS) and the dorsal motor nucleus of the vagus (DMNV). Scale bar, 10 μm.

To determine the involvement of ERK1/2 in m-3M3FBS-induced emesis, we performed immunohistochemistry to detect changes in ERK1/2 phosphorylation expression evoked by m-3M3FBS (50 mg/kg, i. p.). Representative images and statistical graph in Figure 3 demonstrate that following systemic administration of m-3M3FBS, a significant increase in ERK1/2 phosphorylation (*p* = 0.0246) occurred throughout the dorsal vagal complex including the emetic nuclei, AP, NTS and DMNV.

**FIGURE 4:**
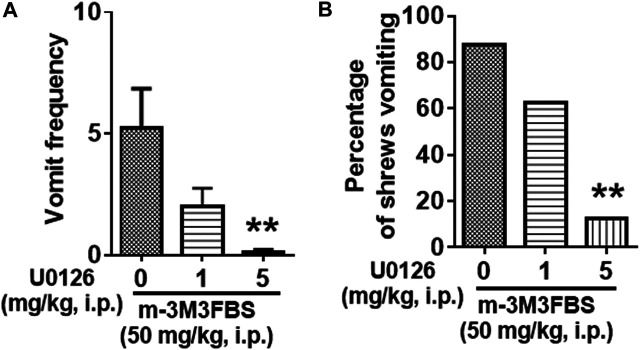
Antiemetic effects of the ERK1/2 inhibitor U0126 against m-3M3FBS-induced emesis. Different groups of shrews were given vehicle or varying doses of U0126 (i.p.) (*n* = 8 shrews per group) 30 min prior to m-3M3FBS (1 mg/kg, i. p.) administration. Emetic parameters were recorded for the next 2 h. **(A)** The frequency of emesis was analyzed with Kruskal-Wallis non-parametric one-way ANOVA followed by Dunnett’s post hoc test and presented as mean ± SEM. **(B)** Percentage of shrews vomiting was analyzed with chi-square test and presented as mean. ***p < 0.001 vs. 0 mg/kg.

Next, we examined the antiemetic potential of the ERK1/2 inhibitor U0126 against m-3M3FBS-induced emesis. The vehicle or U0126 (1 and 5 mg/kg, i. p.) were administered to different groups of shrews 30 min prior to m-3M3FBS (50 mg/kg, i. p.) injection. U0126 pretreatment reduced the frequency of m-3M3FBS-induced vomiting (KW (2, 21) = 11.4, *p* = 0.0034), with a significant reduction in the vomit frequency at its 5 mg/kg dose (*p* = 0.0015) (Figure 4A). The chi-square test showed U0126 pretreatment also reduced the percentage of shrews vomiting (χ^2^ (2, 21) = 9.399, *p* = 0.0091) in response to m-3M3FBS, and achieved significance at 5 mg/kg dose (*p* = 0.0027) (Figure 4B).

### The Protein Kinase C Inhibitor GF109203X Reduces m-3M3FBS-Induced Emesis

**FIGURE 5:**
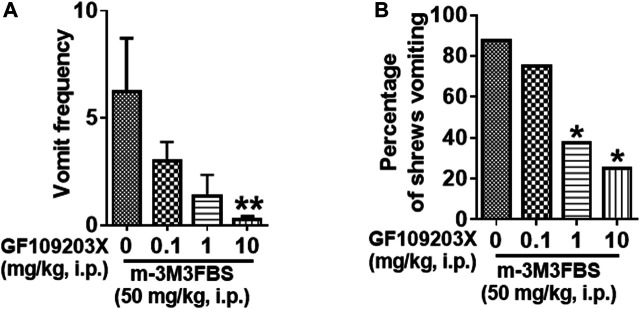
The antiemetic effects of the PKC inhibitor GF109203X against m-3M3FBS-induced emesis. Different groups of least shrews were given an injection of either the corresponding vehicle, or varying doses of GF109203X (i.p.) (*n* = 8 per group) 30 min prior to m-3M3FBS injection (50 mg/kg, i. p.). Emetic parameters were recorded for the next 2 h. **(A)** The frequency of emesis was analyzed with Kruskal-Wallis non-parametric one-way ANOVA followed by Dunnett’s post hoc test and presented as mean ± SEM. **(B)** Percentage of shrews vomiting was analyzed with chi-square test and presented as mean. *p < 0.05, **p < 0.01, ***p < 0.001, ****p < 0.0001 vs. 0 mg/kg.

The antiemetic potential of the PKC inhibitor GF109203X (0.1, 1 and 10 mg/kg) was tested against m-3M3FBS (50 mg/kg, i. p.)-induced vomiting. GF109203X pretreatment reduced both the mean vomit frequency [KW (3, 28) = 12.22, *p* = 0.0067] (Figure 5A) and percentage of shrews vomiting [χ^2^ (3, 28) = 8.635; *p* = 0.0346] (Figure 5B) in response to m-3M3FBS in a dose-dependent manner. Significant reduction in the mean vomit frequency occurred at the 10 mg/kg dose of GF109203X, whereas the percentage of shrews vomiting was significantly reduced from its 1 mg/kg dose.

### Ca^2+^ Channel Modulators Reduce m-3M3FBS-induced Emesis

#### Intracellular Ca^2+^ Channel Inhibitors

**FIGURE 6:**
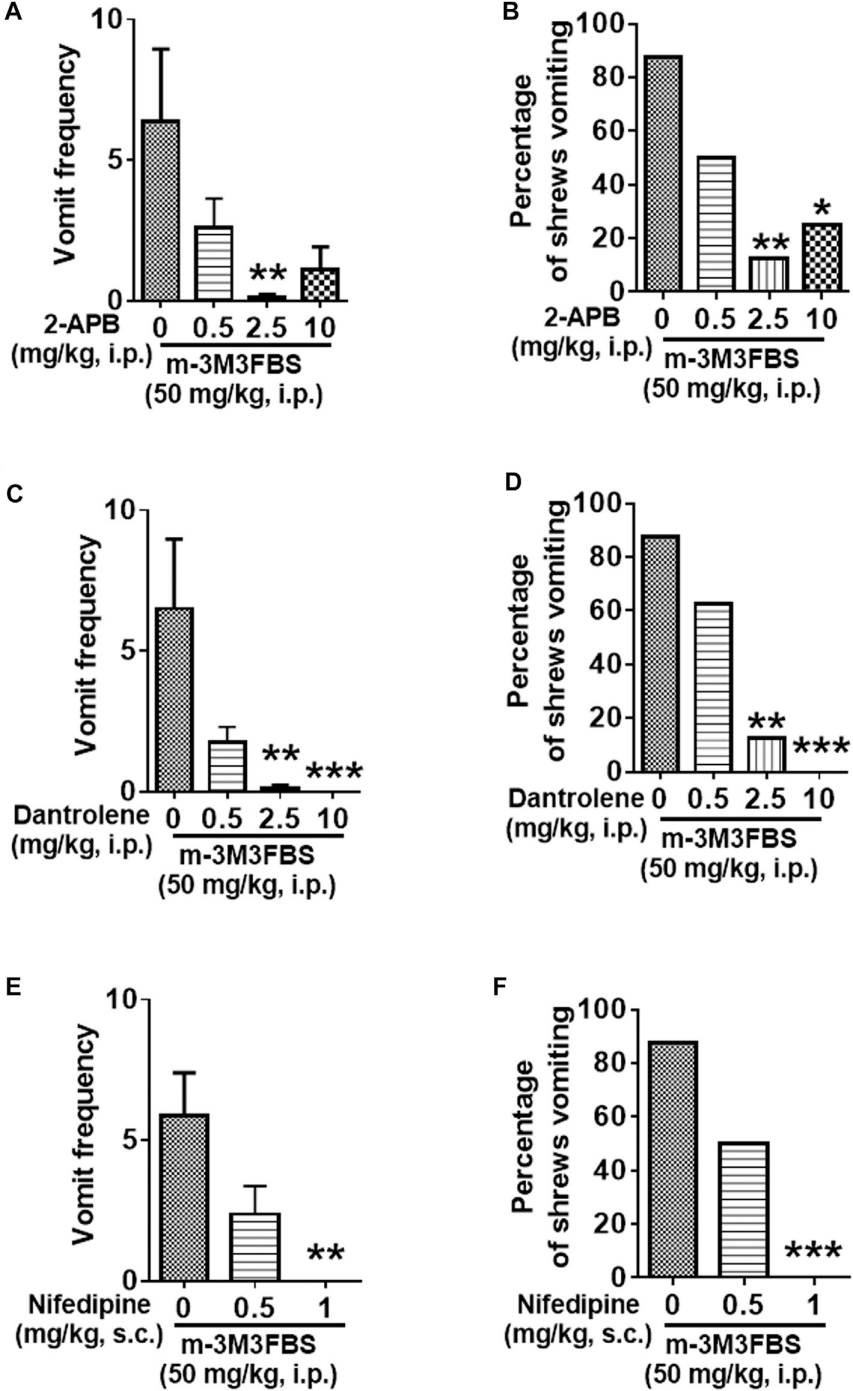
The antiemetic effects of Ca^2+^ channels modulators against m-3M3FBS-induced emesis. Thirty minutes prior to an injection of m-3M3FBS (50 mg/kg, i. p.), different groups of least shrews were given an injection (i.p.) of either the corresponding vehicle, or varying doses of: 1) the IP_3_R antagonist 2-APB (*n* = 8) **(A,B)**, 2) the RyR antagonist dantrolene (*n* = 8 shrews per group) **(C,D)**, or 3) the LTCC inhibitor nifedipine (s.c.) (*n* = 8 shews per group) **(E,F)**. Emetic parameters were recorded for the next 2 h min post m-3M3FBS injection. **(A,C,E)** The frequency of emesis was analyzed with Kruskal-Wallis non-parametric one-way ANOVA followed by Dunnett’s post hoc test and presented as mean ± SEM. **(B,D,F)** The percentage of shrews vomiting was analyzed with chi-square test and presented as mean. *p < 0.05, **p < 0.01, ****p < 0.0001 vs. 0 mg/kg.

We next investigated whether the intracellular Ca^2+^ release channels such as IP_3_Rs and/or RyRs located on the endoplasmic reticulum, are involved in m-3M3FBS-evoked vomiting. IP_3_R blockade with 2-APB (0.5, 2.5, and 10 mg/kg) caused a significant reduction in the frequency of m-3M3FBS-evoked vomiting [KW (3, 28) = 10.69, *p* = 0,135] at its 2.5 (*p* = 0.0076), but not at 10 mg/kg (*p* = 0.0512) dose (Figure 6A). Moreover, 2-APB pretreatment significantly attenuated the percentage of shrews vomiting [χ^2^ (3, 28) = 10.67, *p* = 0.0137] (Figure 6B), with significant reductions occurring at both its 2.5 (*p* = 0.0027) and 10 mg/kg (*p* = 0.0117) doses (Figure 6B). Figure 6C demonstrates that pretreatment with the RyR antagonist dantrolene (0.5, 2.5 and 10 mg/kg, i. p.), significantly and in a dose-dependent manner suppresses the m-3M3FBS -evoked mean vomit frequency [KW (3, 28) = 19.45, *p* = 0.0002] with significant reductions occurring at its 2.5 (*p* = 0.001) and 10 mg/kg doses (*p* = 0.0003). Dantrolene also significantly suppressed the percentage of shrews vomiting in response to m-3M3FBS [χ^2^ (3, 28) = 16.97, *p* = 0.0007], with significant reduction appearing at its 2.5 (*p* = 0.0027) and complete protection at the 10 mg/kg (*p* = 0.0004) doses (Figure 6D).

#### The L-Type Ca^2+^ Channel Inhibitor Nifedipine Suppresses m-3M3FBS-Induced Emesis

Relative to the vehicle-treated control group, nifedipine administration (0.5 and 1 mg/kg, s. c.) 30 min prior to m-3M3FBS (50 mg/kg, i. p.) injection caused dose-dependent suppression of both the mean vomit frequency [KW (2, 21) = 11.89, *p* = 0.0026] and the percentage of shrews vomiting [χ^2^ (2, 21) = 12.42; *p* = 0.0020] in response to m-3M3FBS (Figures 6E,F). Nifedipine at 1 mg/kg completely suppressed m-3M3FBS-evoked vomiting (*p* = 0.0011 for frequency and *p* = 0.0004 for percentage).

### Emetic Receptor Antagonists Reduce m-3M3FBS-Induced Emesis

**FIGURE 7:**
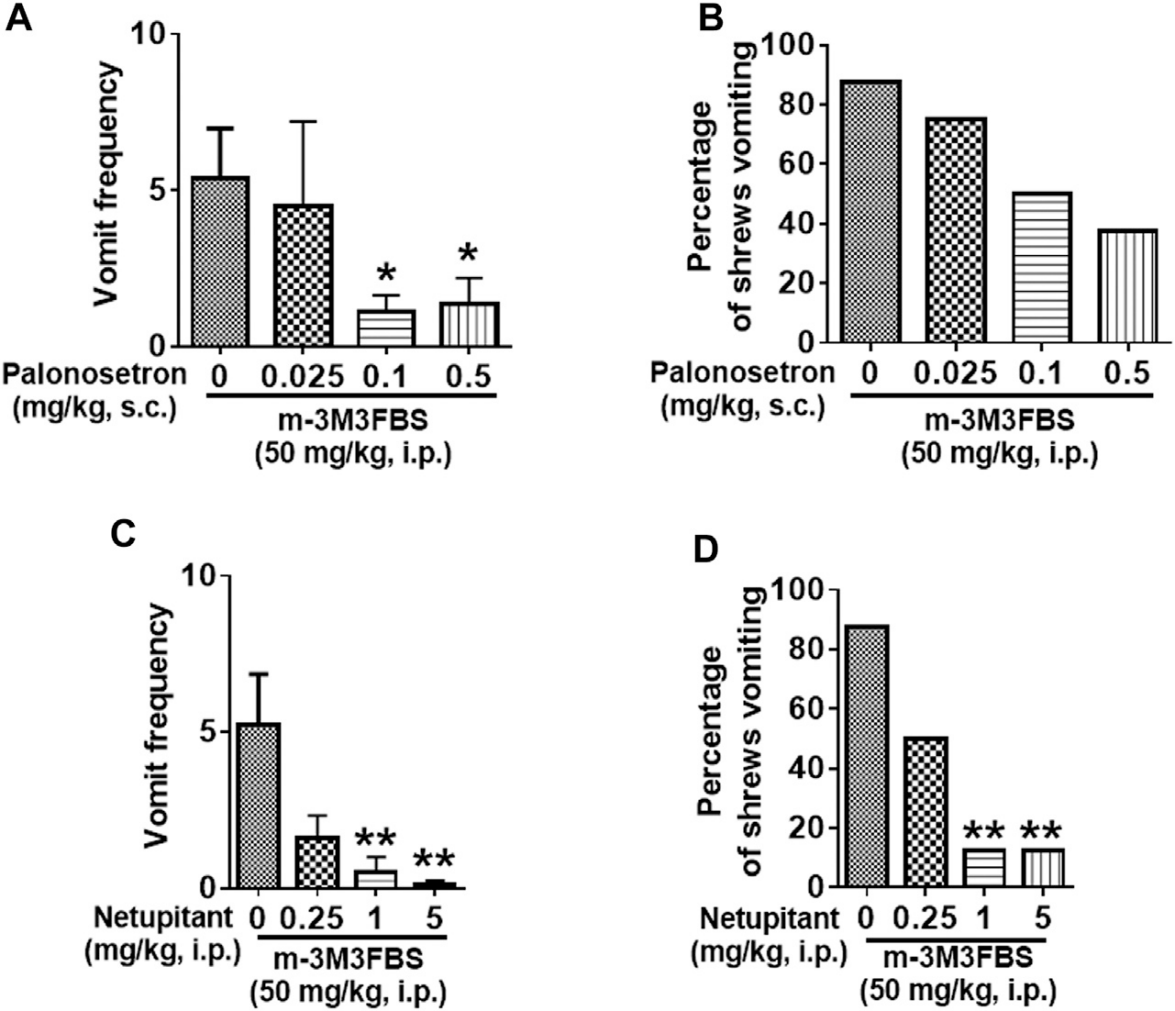
The antiemetic effects of receptor-selective antagonists against m-3M3FBS-induced emesis. Different groups of least shrews were given an injection of either the corresponding vehicles (0 mg/kg), or varying doses of the 5-HT_3_R antagonist palonosetron (s.c.) (*n* = 8 shrews per group) **(A,B)**, or the neurokinin NK_1_R antagonist netupitant (i.p.) (*n* = 8) **(C,D)**, 30 min prior to m-3M3FBS administration (50 mg/kg, i. p.). Emetic parameters were recorded for the next 2 h **(A,C)**. The frequency of emesis was analyzed with Kruskal-Wallis non-parametric one-way ANOVA followed by Dunnett’s post hoc test and presented as mean ± SEM. **(B,D)** Percentage of shrews vomiting was analyzed with chi-square test and presented as mean. *p < 0.05, **p < 0.01 vs. 0 mg/kg.

Different groups of shrews were pretreated with either the 5-HT_3_R antagonist palonosetron (0.025, 0.1, and 0.5 mg/kg, s. c.), the NK_1_R antagonist netupitant (0.25, 1 and 5, i. p.), or their corresponding vehicle, for 30 min prior to m-3M3FBS (50 mg/kg, i. p.) injection. Palonosetron pretreatment reduced the mean vomit frequency [KW (3, 28) = 8.218, *p* = 0.0417] in response to m-3M3FBS (Figure 7A), with significant reduction occurring at its 0.1 (*p* = 0.0460) and 0.5 (*p* = 0.0410) doses. However, palonosetron failed to completely protect shrews from vomiting [χ^2^ (3, 28) = 5.333; *p* = 0.1490] (Figure 7B).

The substance P receptor antagonist netupitant also caused a dose-dependent decrease in the frequency of evoked vomits [(KW (3, 28) = 14.78, *p* = 0.0020)] with significant reductions occurring at its 1 mg/kg (*p* = 0.0038) and 5 mg/kg doses (*p* = 0.0020) (Figure 7C). The chi-square test indicates that the number of shrews vomiting in response to m-3M3FBS was also significantly attenuated by netupitant [(χ^2^ (3, 28) = 12.83, *p* = 0.0050)] (Figure 7D) with ∼80% protection at 1 mg/kg and 5 mg/kg doses (*p* = 0.0027).

### The Antiemetic Potential of the PLC Inhibitor U73122

**FIGURE 8:**
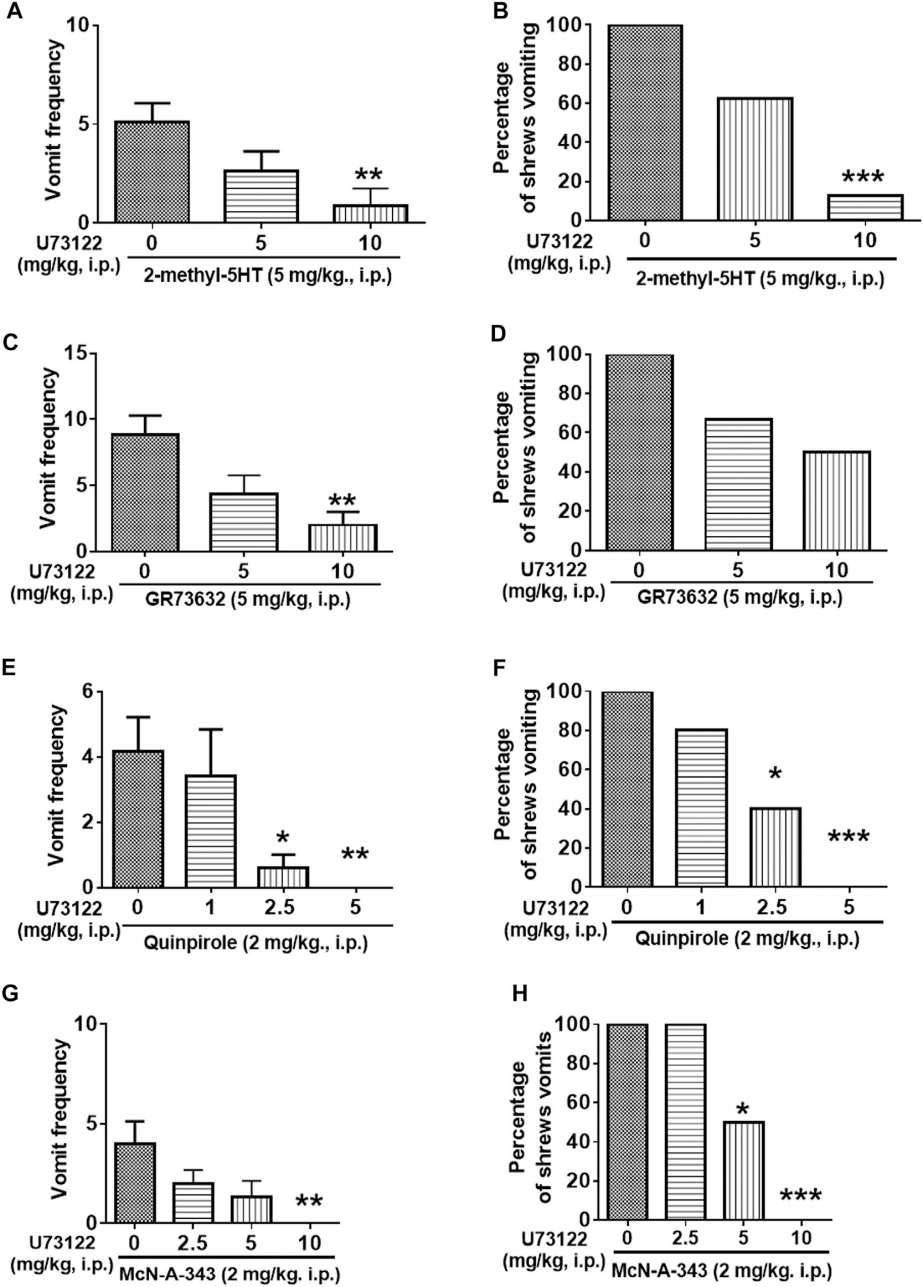
The antiemetic effects of the PLC inhibitor U73122 against vomiting evoked by diverse receptor-selective emetogens. Varying doses of U73122 (i.p.) were injected to different groups of shrews 30 min prior to an injection of a fully effective emetic dose of selective serotonin 5-HT_3_ receptor agonist 2-Methyl-5-HT (5 mg/kg, i. p., *n* = 8) **(A,B)**, the selective neurokinin NK_1_ receptor agonist GR73632 (5 mg/kg, i. p., *n* = 6) **(C,D)**, the dopamine D_2/3_ receptor preferring agonist quinpirole (2 mg/kg, i. p., *n* = 5) **(E,F)**, or the muscarinic M_1_ receptor agonist McN-A-343 (2 mg/kg, i. p., *n* = 6) **(G,H)**. Emetic parameters were recorded for the next 30 min **(A,C,E,G)** The frequency of emesis was analyzed with Kruskal-Wallis non-parametric one-way ANOVA followed by Dunnett’s post hoc test and presented as mean ± SEM. **(B,D,F,H)** The percentage of shrews vomiting was analyzed with chi-square test and presented as the mean. *p < 0.05, **p < 0.01, ***p < 0.001, ****p < 0.0001 vs. 0 mg/kg (controls pretreated with vehicle of U73122).

Figure 8 demonstrates the antiemetic potential of U73122 against vomiting evoked by diverse emetic receptor agonists. In fact, pretreatment with U73122 (5 and 10 mg/kg), reduced both the frequency (KW (2, 21) = 8.768, *p* = 0.0125) and percentage (χ^2^ (2, 21) = 12.69; *p* = 0.0018) of shrews vomiting in response to the administration of the 5-HT_3_R-selective agonist, 2-Methyl-5-HT (5 mg/kg, i. p.) (Figures 8A,B). A significant reduction in vomit frequency occurred at its 10 mg/kg dose (*p* = 0.0062). In addition, the percentage of shrews vomiting was reduced by 87.5% at 10 mg/kg dose (*p* = 0.0004). As shown in Figures 8C,D, U73122 (5 and 10 mg/kg) also caused a dose-dependent decrease in the mean frequency of vomits induced by the NK_1_R selective agonist GR73632 (KW (2, 15) = 8.693, *p* = 0.0076) with a significant reduction at its 10 mg/kg dose (*p* = 0.0072), but no significant decrease (χ^2^ (2, 15) = 3.877; *p* = 0.1439) in the percentage of animals vomiting was observed.

Furthermore, the antiemetic effect of U73122 (1, 2.5 and 5 mg/kg) was also assessed against vomiting caused by the dopamine D_2/3_R agonist, quinpirole (2 mg/kg, i. p.) (Figures 8E,F). The mean frequency of quinpirole-induced emesis (KW (3, 16) = 12.21, *p* = 0.0067) was significantly reduced with complete suppression at its 5 mg/kg dose (*p* = 0.0060). Significant decrease (χ^2^ (3, 16) = 11.92; *p* = 0.0077) in the percentage of animals vomiting were noted at its 2.5 (60%; *p* = 0.0261) and complete protection at 5 mg/kg dose (100%; *p* = 0.0009).

Next, the antiemetic effect of varying doses of U73122 was tested against vomiting caused by the muscarinic M_1_R agonist, McN-A-343 (2 mg/kg, i. p.) (Figures 8G,H). U73122 pretreatment (2.5, 5 and 10 mg/kg) also significantly reduced the frequency of McN-A-343-induced emesis (KW (3, 20) = 12.97, *p* = 0.0047) in a dose-dependent manner with significant reduction at 10 mg/kg dose (*p* = 0.0015). The percentage of shrews vomiting was also reduced in a dose-dependent fashion (χ^2^ (3, 20) = 14.4; *p* = 0.0024) with significant reductions at 5 (50%; *p* = 0.0455) and complete protection at its 10 mg/kg dose (100%; *p* = 0.0005).

**FIGURE 9:**
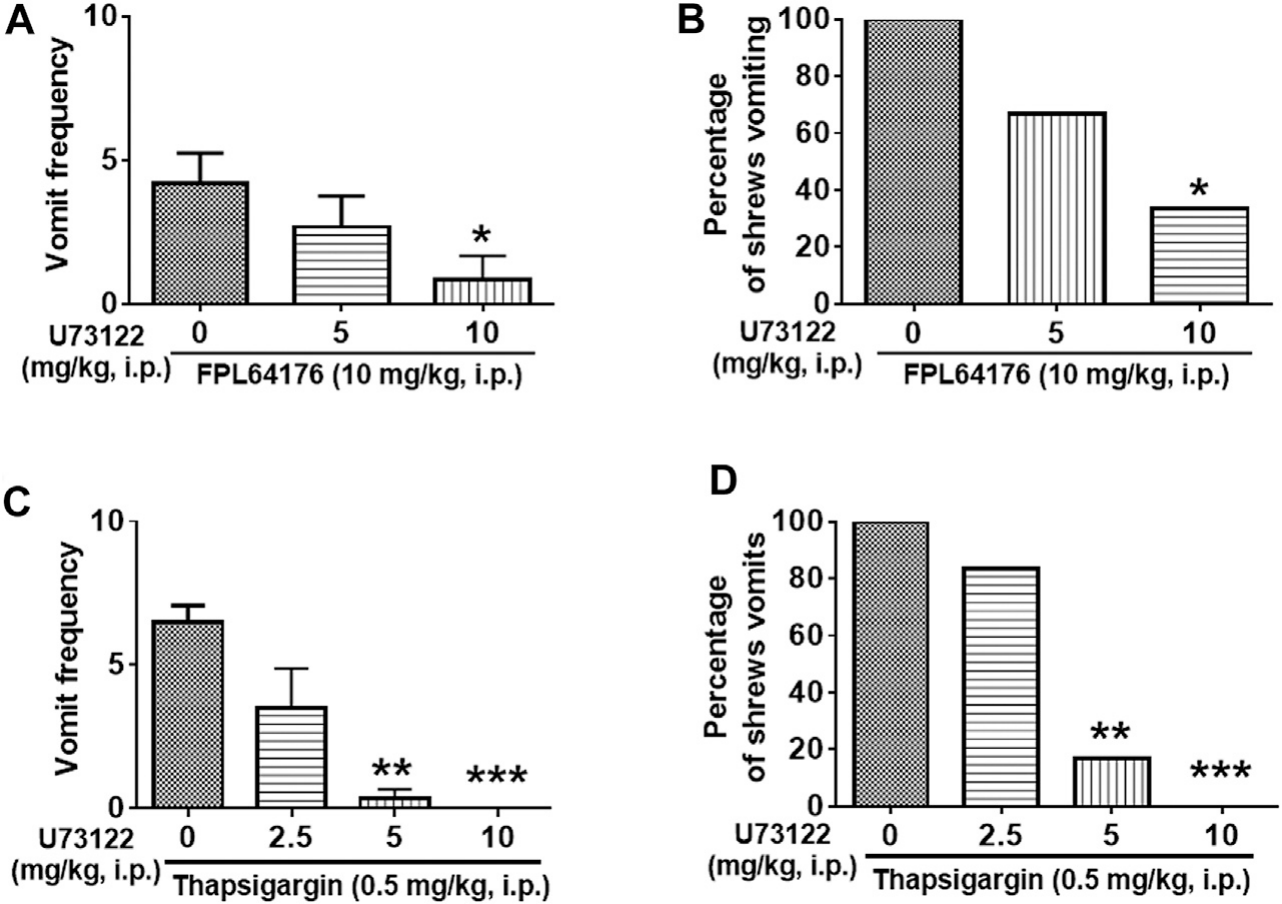
The antiemetic effects of the selective plc inhibitor U73122 against vomiting caused by Ca^2+^ channel regulators: the LTCC agonist FPL64176 and the SERCA inhibitor thapsigargin. Varying doses of U73122 (i.p.) were injected to different groups of shrews 30 min prior to an injection of a fully effective emetic dose of FPL64176 (10 mg/kg, i. p., *n* = 6) **(A,B)**, or thapsigargin (0.5 mg/kg, i. p., *n* = 6) **(C,D)**. Emetic parameters were recorded for the next 30 min **(A,C)** The frequency of emesis was analyzed with Kruskal-Wallis non-parametric one-way ANOVA followed by Dunnett’s post hoc test and presented as mean ± SEM. **(B,D)** Percentage of shrews vomiting was analyzed with chi-square test and presented as the mean. *p < 0.05, **p < 0.01, ***p < 0.001 vs. 0 mg/kg.

We then investigated the antiemetic potential of U73122 against vomiting evoked by Ca^2+^ channel regulators, such as the selective LTCC agonist FPL64176 (10 mg/kg, i. p.) and the SERCA inhibitor thapsigargin (0.5 mg/kg, i. p.) (Figure 9). U73122 (5 and 10 mg/kg) significantly attenuated the mean frequency of FPL64176-induced vomiting in a dose-dependent manner (KW (2, 15) = 6.195, *p* = 0.0384) with a significant reduction at 10 mg/kg dose (*p* = 0.0257) (Figures 9A,B). In addition, the percentage of shrews vomiting in response to FPL64176 was also suppressed by U73122 (χ^2^ (2, 15) = 6; *p* = 0.0498) with a 66.7% reduction at its 10 mg/kg (*p* = 0.0143). Figures 9C,D show that U73122 (2.5, 5 and 10 mg/kg) dose-dependently suppressed vomiting caused by thapsigargin (0.5 mg/kg, i. p.). The frequency of thapsigargin-induced emesis (KW (3, 20) = 18.09, *p* = 0.0004) was significantly reduced at 5 (*p* = 0.0023) and complete reduction occurred at its 10 mg/kg dose (*p* = 0.0007). Likewise, significant decreases (χ^2^ (3, 20) = 17.33; *p* = 0.0006) in the percentage of animals vomiting were also noted at its 5 (83.3%; *p* = 0.0034) and complete protection at the 10 mg/kg dose (100%; *p* = 0.0005).

## Discussion

### Significance of This Study

Phospholipase C (PLC) activation is a crucial component in cellular signaling arising from the activation of diverse GPCRs, the largest family of cell membrane-bound receptors, and plays critical roles in signal transduction (Dorsam and Gutkind, 2007). It has been established that a wide variety of GPCRs mediate vomiting when stimulated by endogenous stimuli such as neuropeptides (e.g., substance P) (Carpenter et al., 1984), biogenic amines (e.g., histamine) (Bhargava and Dixit, 1968), lipids (e.g. prostaglandins) (Kan et al., 2006) as well as diverse pharmaceutical agents (Darmani et al., 2014; Zhong et al., 2018). However, to date, scant evidence exists to support a role for PLC in the emetic processes. In this study we demonstrated that a PLC activator, m-3M3FBS, induces vomiting in the least shrew model of emesis, with a mean maximal frequency (6.25 vomits ±2.6) of vomits occurring at its 50 mg/kg (i.p.) dose in up to 90% tested shrews. Because of solubility issues, we could not test a larger dose of m-3M3FBS. In addition, the PLC inhibitor U73122 not only suppressed m-3M3FBS-evoked vomiting, but also vomits induced by selective agonists of diverse emetic receptors including serotonin 5-HT_3_-, neurokinin NK_1_-, dopamine D_2/3_-, and muscarinic M_1_-receptors. Thus, the present study provides direct evidence for PLC participation in emesis. The major limitation of this study is that the molecular mechanisms by which PLC activation-mediates vomiting remain to be deciphered. In the following sections, we attempt to discuss the potential mechanisms by which downstream signaling molecules following administration of the PLC activator m-3M3FBS may contribute to the evoked emesis.

### Potential Mechanisms in PLC Activator-Induced Emesis

As we discussed in the introduction section, Ca^2+^, ERK1/2 and PKC are important downstream effectors in PLC-dependent signal transduction. In the current study, vomiting induced by the PLC activator m-3M3FBS was followed by increased expression of c-Fos and ERK1/2 phosphorylation in the brainstem dorsal vagal complex, indicating central activation of emetic nuclei (the area postrema, the NTS and DMNV) following systemic administration of m-3M3FBS. The c-Fos expression in the enteric nervous system of the jejunum showed a weak response upon m-3M3FBS-induced vomiting, whereas the statistics analysis showed no significant difference (*p* = 0.077) between vehicle controls and m-3M3FBS-administed shrews. Since the brainstem area postrema lacks a complete blood-brain barrier (Wickham, 2020), circulating drugs may directly act on this emetic region, which would then sends output to the NTS for integration, and ultimately sensory signals are conveyed to the DMNV which mediates the emetic motor signals to gastrointestinal tract during the vomiting process (Hornby, 2001; Darmani and Ray, 2009; Bashashati and McCallum, 2014). According to published literature, m-3M3FBS can directly activate different PLC isoforms in various cell types, which can lead to generation of both IP_3_ as well as subsequent increase in intracellular Ca^2+^ (Bae et al., 2003). Moreover, different PLC isoforms (PLC_β_, PLC_γ_, PLC_δ_, and PLC_ε_) can also regulate the ERK1/2 signaling cascade (Owusu Obeng et al., 2020).

In this study the emetic action of m-3M3FBS appears to be sensitive to inhibitors of ERK1/2 (U0126), PKC (GF109203X), and IP_3_R (2-APB), which are in line with current understanding that DAG, IP_3_, and Ca^2+^ mobilization, are involved in PLC-dependent cell signaling. In addition, our published findings implicate ERK1/2 phosphorylation in the brainstem DVC as a common emetic signal elicited by systemic administration (i.p.) of diverse emetogens including the: 1) NK_1_ receptor agonist GR73632 (5 mg/kg), 2) LTCC activator FPL64176 (10 mg/kg), 3) the SERCA inhibitor and thus an intracellular Ca^2+^ signaling amplifier, thapsigargin (0.5 mg/kg), 4) the 5-HT_3_ receptor agonist 2-Methyl-5-HT (5 mg/kg) (Zhong et al., 2014; Zhong et al., 2016; Zhong et al., 2018; Zhong et al., 2019), 5) chemotherapeutic agent cisplatin (10 mg/kg, i. p.) (Darmani et al., 2015), and 6) Akt inhibitor MK-2206 (10 mg/kg, i. p.) (Zhong et al., 2021). Moreover, ERK1/2 inhibitors such as PD98059 or U0126, exert substantial or partial antiemetic effects against vomiting-evoked by the above discussed emetogens except cisplatin.

PKC phosphorylation in least shrew brainstem has also been found to be associated with vomiting caused by several emetogens including cisplatin (Darmani et al., 2013; Darmani et al., 2015), FPL64176 and GR73632 (Zhong et al., 2018; Zhong et al., 2019). Furthermore, the PKC inhibitor GF109203X can suppress the vomiting evoked by either FPL64176 or GR73632 (Zhong et al., 2018; Zhong et al., 2019).

Both IP_3_R and RyR contribute to increased intracellular Ca^2+^ concentration following PLC activation (Kim et al., 2015). In this study, inhibitors of both IP_3_R (2-APB) and RyR (dantrolene) displayed antiemetic efficacy against m-3M3FBS-induced vomiting, suggesting the involvement of these intracellular endoplasmic reticulum Ca^2+^ release channels in emesis. Whether the antiemetic role of 2-APB and dantrolene contribute to inhibition of intracellular Ca^2+^ release needs further Ca^2+^ imaging studies on cells or brain slices of an emetic species. Previously we have found that dantrolene and 2-APB exhibit differential antiemetic efficacy against vomiting elicited by several emetogens, such as the 5-HT_3_R agonist 2-Methyl-5HT (Zhong et al., 2014), FPL64176 (Zhong et al., 2018), thapsigargin (Zhong et al., 2016), and NK_1_R agonist GR73632 (Zhong et al., 2014; Zhong et al., 2016; Zhong et al., 2018; Zhong et al., 2019). Therefore, RyRs and IP_3_Rs ion-channels may be differentially regulated by different emetogens and may be potential targets for emesis prevention. 2-APB has often been used as an IP_3_R inhibitor, but other studies suggest it also acts as a store-operated Ca^2+^ entry inhibitor (Hofer et al., 2013). This non-selectivity of 2-APB may help explain our current findings in that the reduction in frequency of m-3M3FBS-induced vomiting by 2-APB is significant at its 2.5 mg/kg dosage, but not at the10 mg/kg dosage.

The complete blockade of m-3M3FBS-induced vomiting by LTCC inhibitor nifedipine is not surprising. In the least shrew emesis model, nifedipine exhibits broad-spectrum antiemetic efficacy against vomiting evoked by a myriad of emetogens including agonists of the LTCC (FPL64176), 5-HT_3_- (e.g., 5-HT or 2-Me-5-HT), NK_1_- (GR73632), dopamine D_2/3_- (apomorphine or quinpirole), muscarinic M_1_- (McN-A-343) receptors (Darmani et al., 2014). Ca^2+^ mobilization is involved in both triggering neurotransmitter release coupled with receptor activation, as well as post-receptor excitation-contraction coupling (Zuccotti et al., 2011), which can be an important aspect of vomit induction, and has been further discussed in one of our reviews (Zhong et al., 2017). The mechanism underlying the antiemetic potential of Ca^2+^ signaling inhibitors may be closely related to inhibition of Ca^2+^ mobilization and emetic receptor-mediated downstream signaling pathways involving key molecules, such as PKA, PKC, ERK1/2, and Ca^2+^/calmodulin-dependent protein kinase II (Zhong et al., 2017).

m-3M3FB-evoked emesis is also sensitive to antagonists of both 5-HT_3_R and NK_1_R (palonosetron and netupitant, respectively). It remains to be determined whether the PLC activator m-3M3FBS stimulates release of 5-HT or substance P, or both, which would subsequently activate corresponding serotonin 5-HT_3_R and/or neurokinin NK_1_R to evoke vomiting, which our accompanying antagonist studies imply. To the best of our knowledge, there is no published literature to indicate PLC activation by m-3M3FBS would cause release of such emetic mediators. However, it is known that the PLC inhibitor U73122 inhibits lipopolysaccharide-induced prostaglandin E_2_ production in mice, a process attributed to the inhibition of PLC pathway (Hou et al., 2004). Moreover, lipopolysaccharide is a potent emetogen in pigs (Girod et al., 2000).

### Antiemetic-Effects of the PLC Inhibitor U73122

U73122 can selectively inhibit the PLC-dependent signaling process, and thus has proven useful in evaluating PLC-dependent cell activation both *in vitro* and *in vivo* when administered i. p. or intravenously (Hou et al., 2004). In the present study, U73122 suppressed vomiting evoked by the PLC activator m-3M3FBS, or via the activation of key emetic receptors (serotonin 5-HT_3_, neurokinin NK_1_, dopamine D_2/3_, muscarinic M_1_) induced by their corresponding specific agonists (2-Methyl-5-HT, GR73632, quinpirole, McN-A-343, respectively) and Ca^2+^ signaling amplifiers (FPL64176 and thapsigargin) (Darmani et al., 2014; Zhong et al., 2016). U73122 is a PLC isoform-nonspecific inhibitor which also attenuates stimulus-evoked intracellular Ca^2+^ accumulation as well as ERK1/2 phosphorylation in cellular studies (Shen et al., 2013). Therefore, the mechanism underlying the antiemetic potential of U73122 could be interpreted via inhibition of Ca^2+^ mobilization and ERK1/2 signaling, both of which play important roles in the vomiting-mediated by various emetic receptors as demonstrated in our previous studies (Zhong et al., 2017). Another potential explanation could be stimulation of emetic receptors (e.g., 5-HT_3_R, LTCC) may activate PLC through Ca^2+^ mobilization (Thore et al., 2005).

The tested antiemetic doses of U73122 may nonspecifically attenuate vomiting via a general decrease in locomotor activity. Thus, we investigated the effect of U73122 on locomotor activity parameters of least shrews using a computerized video tracking, motion analysis and behavior recognition system (EthoVision) as described in our published studies (Darmani, 2002). The tested antiemetic doses of U73122 in this study did not affect either the velocity or distance travelled by the shrews (Supplementary Figure S1). Thus, inhibition of motor activity per se does not contribute to the antiemetic potential of U73122.

### Anti-Nauseous Potential of the PLC Inhibitor U73122

Per our introduction section, in addition to the described limitation of the current study concerning molecular mechanisms by which PLC activation-mediates vomiting, the potential anti-nauseous activity of U73122 in the least shrew remains to be established. Indeed, published anatomical and pharmacological data imply several higher brain structures (e.g., amygdala, cortex), as well as neurotransmitters (e.g., cholinergic system, histaminergic), their corresponding receptors, and cellular processes (e.g., expression of immediate early genes, kinase signaling pathway) are probably involved in nausea (Welzl, 2001). As suggested by our current emesis/antiemesis findings, PLC activators and inhibitors would probably exert similar nauseous/anti-nauseous outcomes which would need to be investigated.

## Conclusion and Prospects

Taken together, our findings demonstrate that when administered in the least shrew systematically, the PLC activator m-3M3FBS behaves as a proemetic agent. The evoked vomiting is accompanied by central activation of emetic loci as indicated by the evoked c-Fos expression and ERK1/2 phosphorylation in the brainstem dorsal vagal complex. The induced vomiting is sensitive to inhibitors of Ca^2+^ signaling, ERK1/2, PKC as well as emetic receptors (serotonin 5-HT_3_ and neurokinin NK_1_). Furthermore, the PLC inhibitor U73122 is efficacious in reducing the emetic effects of m-3M3FBS as well as agonists of emetic GPCRs, suggesting PLC serves as an upstream activator of the cellular responses to such emetogens.

Furthermore, inhibitors targeting PLC signaling, especially PLC itself, and its downstream effectors (e.g., Ca^2+^, IP_3_, DAG, PKC and ERK1/2), may provide antiemetic efficacy in patients. Our long-term goal is to find new classes of antiemetic/antinausea drugs which could concurrently prevent both chemotherapy-induced nausea and vomiting (CINV) in cancer patients. Targeting common downstream intracellular emetic signals may provide new avenues for development of much-needed drugs to suppress both gastrointestinal side-effects of cisplatin-type chemotherapeutics.

## Data Availability

The original contributions presented in the study are included in the article/Supplementary Material, further inquiries can be directed to the corresponding author.

## Abbreviations

PLC, phospholipase C; AP, area postrema; NTS, nucleus tractus solitarius; DMNV, dorsal motor nucleus of the vagus; GPCRs, G protein-coupled receptors; 5-HT3R, Serotonergic type 3 receptor; NK1R, neurokinin 1 receptor; IP3R, inositol-1, 4, 5-triphosphate receptor; DAG, diacylglycerol; PKC, protein kinase C; ERK1/2, extracellular signal-regulated protein kinase1/2; LTCC, L-type Ca2+ channel; RyR, ryanodine receptor; i. p., intraperitoneally; s. c., subcutaneously; 5-HT, serotonin; SERCA, sarco/endoplasmic reticulum Ca2+-ATPase; CINV, chemotherapy-induced nausea and vomiting.

## References

[B1] Babic T. Browning K. N. (2014). The Role of Vagal Neurocircuits in the Regulation of Nausea and Vomiting. Eur. J. Pharmacol. 722, 38–47. 10.1016/j.ejphar.2013.08.047 24184670 PMC3893663

[B2] Bae Y. S. Lee T. G. Park J. C. Hur J. H. Kim Y. Heo K. (2003). Identification of a Compound that Directly Stimulates Phospholipase C Activity. Mol. Pharmacol. 63 (5), 1043–1050. 10.1124/mol.63.5.1043 12695532

[B3] Bashashati M. McCallum R. W. (2014). Neurochemical Mechanisms and Pharmacologic Strategies in Managing Nausea and Vomiting Related to Cyclic Vomiting Syndrome and Other Gastrointestinal Disorders. Eur. J. Pharmacol. 722, 79–94. 10.1016/j.ejphar.2013.09.075 24161560

[B4] Beleslin D. B. Nedelkovski V. (1988). Emesis Induced by 4-(m-Chlorophenylcarbamoyloxy)-2-Butynyltrimethylammonium Chloride (McN-A-343): Evidence for a Predominant central Muscarinic M1 Mediation. Neuropharmacology 27 (9), 949–956. 10.1016/0028-3908(88)90123-2 2460797

[B5] Bhargava K. P. Dixit K. S. (1968). Role of the Chemoreceptor Trigger Zone in Histamine-Induced Emesis. Br. J. Pharmacol. 34 (3), 508–513. 10.1111/j.1476-5381.1968.tb08479.x 4387255 PMC1703476

[B6] Carpenter D. O. Briggs D. B. Strominger N. (1984). Behavioral and Electrophysiological Studies of Peptide-Induced Emesis in Dogs. Fed. Proc. 43 (15), 2952–2954. 6149958

[B7] Darmani N. A. Dey D. Chebolu S. Amos B. Kandpal R. Alkam T. (2013). Cisplatin Causes Over-expression of Tachykinin NK(1) Receptors and Increases ERK1/2- and PKA- Phosphorylation during Peak Immediate- and Delayed-phase Emesis in the Least Shrew (Cryptotis Parva) Brainstem. Eur. J. Pharmacol. 698 (1-3), 161–169. 10.1016/j.ejphar.2012.09.008 23001014

[B8] Darmani N. A. Ray A. P. (2009). Evidence for a Re-evaluation of the Neurochemical and Anatomical Bases of Chemotherapy-Induced Vomiting. Chem. Rev. 109 (7), 3158–3199. 10.1021/cr900117p 19522506

[B9] Darmani N. A. (1998). Serotonin 5-HT3 Receptor Antagonists Prevent Cisplatin-Induced Emesis in Cryptotis Parva: a New Experimental Model of Emesis. J. Neural Transm. (Vienna) 105 (10-12), 1143–1154. 10.1007/s007020050118 9928884

[B10] Darmani N. A. (2002). The Potent Emetogenic Effects of the Endocannabinoid, 2-AG (2-arachidonoylglycerol) Are Blocked by delta(9)-tetrahydrocannabinol and Other Cannnabinoids. J. Pharmacol. Exp. Ther. 300 (1), 34–42. 10.1124/jpet.300.1.34 11752094

[B11] Darmani N. A. Zhong W. Chebolu S. Mercadante F. (2015). Differential and Additive Suppressive Effects of 5-HT3 (Palonosetron)- and NK1 (Netupitant)-receptor Antagonists on Cisplatin-Induced Vomiting and ERK1/2, PKA and PKC Activation. Pharmacol. Biochem. Behav. 131, 104–111. 10.1016/j.pbb.2015.02.010 25687374

[B12] Darmani N. A. Zhong W. Chebolu S. Vaezi M. Alkam T. (2014). Broad-spectrum Antiemetic Potential of the L-type Calcium Channel Antagonist Nifedipine and Evidence for its Additive Antiemetic Interaction with the 5-HT(3) Receptor Antagonist Palonosetron in the Least Shrew (Cryptotis Parva). Eur. J. Pharmacol. 722, 2–12. 10.1016/j.ejphar.2013.08.052 24513517

[B13] Domingues M. F. de Assis D. R. Belo C. A. D. da Costa J. C. (2018). Peptide YY (3-36) Modulates Intracellular Calcium through Activation of the Phosphatidylinositol Pathway in Hippocampal Neurons. Neuropeptides 67, 1–8. 10.1016/j.npep.2017.11.003 29157865

[B14] Dorsam R. T. Gutkind J. S. (2007). G-protein-coupled Receptors and Cancer. Nat. Rev. Cancer 7 (2), 79–94. 10.1038/nrc2069 17251915

[B15] Frégeau M. O. Carrier M. Guillemette G. (2013). Mechanism of Dopamine D2 Receptor-Induced Ca(2+) Release in PC-12 Cells. Cell Signal 25 (12), 2871–2877. 10.1016/j.cellsig.2013.08.021 24055909

[B16] Girod V. Bouvier M. Grélot L. (2000). Characterization of Lipopolysaccharide-Induced Emesis in Conscious Piglets: Effects of Cervical Vagotomy, Cyclooxygenase Inhibitors and a 5-HT(3) Receptor Antagonist. Neuropharmacology 39 (12), 2329–2335. 10.1016/s0028-3908(00)00091-5 10974316

[B17] Goldsmith Z. G. Dhanasekaran D. N. (2007). G Protein Regulation of MAPK Networks. Oncogene 26 (22), 3122–3142. 10.1038/sj.onc.1210407 17496911

[B18] Grouzmann E. Meyer C. Bürki E. Brunner H. (2001). Neuropeptide Y Y2 Receptor Signalling Mechanisms in the Human Glioblastoma Cell Line LN319. Peptides 22 (3), 379–386. 10.1016/s0196-9781(01)00344-8 11287092

[B19] Hagbom M. Istrate C. Engblom D. Karlsson T. Rodriguez-Diaz J. Buesa J. (2011). Rotavirus Stimulates Release of Serotonin (5-HT) from Human Enterochromaffin Cells and Activates Brain Structures Involved in Nausea and Vomiting. Plos Pathog. 7 (7), e1002115. 10.1371/journal.ppat.1002115 21779163 PMC3136449

[B20] Hauss P. Mazerolles F. Hivroz C. Lecomte O. Barbat C. Fischer A. (1993). GF109203X, a Specific PKC Inhibitor, Abrogates Anti-CD3 Antibody-Induced Upregulation of CD4+ T Cell Adhesion to B Cells. Cell Immunol 150 (2), 439–446. 10.1006/cimm.1993.1211 8103710

[B21] Hille B. Dickson E. J. Kruse M. Vivas O. Suh B. C. (2015). Phosphoinositides Regulate Ion Channels. Biochim. Biophys. Acta 1851 (6), 844–856. 10.1016/j.bbalip.2014.09.010 25241941 PMC4364932

[B22] Hofer A. Kovacs G. Zappatini A. Leuenberger M. Hediger M. A. Lochner M. (2013). Design, Synthesis and Pharmacological Characterization of Analogs of 2-aminoethyl Diphenylborinate (2-APB), a Known Store-Operated Calcium Channel Blocker, for Inhibition of TRPV6-Mediated Calcium Transport. Bioorg. Med. Chem. 21 (11), 3202–3213. 10.1016/j.bmc.2013.03.037 23602525

[B23] Hornby P. J. (2001). Central Neurocircuitry Associated with Emesis. Am. J. Med. 111 Suppl 8A, 106S–112S. 10.1016/s0002-9343(01)00849-x 11749934

[B24] Hotokezaka H. Sakai E. Kanaoka K. Saito K. Matsuo K. Kitaura H. (2002). U0126 and PD98059, Specific Inhibitors of MEK, Accelerate Differentiation of RAW264.7 Cells into Osteoclast-like Cells. J. Biol. Chem. 277 (49), 47366–47372. 10.1074/jbc.M208284200 12237315

[B25] Hou C. Kirchner T. Singer M. Matheis M. Argentieri D. Cavender D. (2004). *In Vivo* activity of a Phospholipase C Inhibitor, 1-(6-((17beta-3-Methoxyestra-1,3,5(10)-Trien-17-Yl)amino)hexyl)-1h-Pyrrole-2,5-Dione (U73122), in Acute and Chronic Inflammatory Reactions. J. Pharmacol. Exp. Ther. 309 (2), 697–704. 10.1124/jpet.103.060574 14730005

[B26] Ilyaskina O. S. Lemoine H. Bünemann M. (2018). Lifetime of Muscarinic Receptor-G-Protein Complexes Determines Coupling Efficiency and G-Protein Subtype Selectivity. Proc. Natl. Acad. Sci. U S A. 115 (19), 5016–5021. 10.1073/pnas.1715751115 29686069 PMC5948956

[B27] Iwasaki Y. Kakei M. Nakabayashi H. Ayush E. A. Hirano-Kodaira M. Maejima Y. (2013). Pancreatic Polypeptide and Peptide YY3-36 Induce Ca2+ Signaling in Nodose Ganglion Neurons. Neuropeptides 47 (1), 19–23. 10.1016/j.npep.2012.07.006 22944736

[B28] Javid H. Mohammadi F. Zahiri E. Hashemy S. I. (2019). The Emerging Role of Substance P/neurokinin-1 Receptor Signaling Pathways in Growth and Development of Tumor Cells. J. Physiol. Biochem. 75 (4), 415–421. 10.1007/s13105-019-00697-1 31372898

[B29] Jijón-Lorenzo R. Caballero-Florán I. H. Recillas-Morales S. Cortés H. Avalos-Fuentes J. A. Paz-Bermúdez F. J. (2018). Presynaptic Dopamine D2 Receptors Modulate [ 3 H]GABA Release at StriatoPallidal Terminals via Activation of PLC → IP3 → Calcineurin and Inhibition of AC → cAMP → PKA Signaling Cascades. Neuroscience 372, 74–86. 10.1016/j.neuroscience.2017.12.041 29292080

[B30] Jo M. Jung S. T. (2016). Engineering Therapeutic Antibodies Targeting G-Protein-Coupled Receptors. Exp. Mol. Med. 48, e207. 10.1038/emm.2015.105 26846450 PMC4892866

[B31] Joshi S. Lee J. W. Wong Y. H. (1999). Stimulation of Phospholipase C by the Cloned Mu, delta and Kappa Opioid Receptors via Chimeric G Alpha(q) Mutants. Eur. J. Neurosci. 11 (2), 383–388. 10.1046/j.1460-9568.1999.00442.x 10051738

[B32] Kan K. K. Rudd J. A. Wai M. K. (2006). Differential Action of Anti-emetic Drugs on Defecation and Emesis Induced by Prostaglandin E2 in the Ferret. Eur. J. Pharmacol. 544 (1-3), 153–159. 10.1016/j.ejphar.2006.06.034 16844111

[B33] Kawakami T. Xiao W. (2013). Phospholipase C-β in Immune Cells. Adv. Biol. Regul. 53 (3), 249–257. 10.1016/j.jbior.2013.08.001 23981313 PMC4324595

[B34] Kim S. D. Kim H. J. Shim J. W. Lee H. Y. Lee S. K. Kwon S. (2012). Phospholipase C Activator m-3M3FBS Protects against Morbidity and Mortality Associated with Sepsis. J. Immunol. 189 (4), 2000–2005. 10.4049/jimmunol.1200635 22798676

[B35] Kim S. W. Cho T. Lee S. (2015). Phospholipase C-Β1 Hypofunction in the Pathogenesis of Schizophrenia. Front. Psychiatry 6, 159. 10.3389/fpsyt.2015.00159 26635636 PMC4648068

[B36] Krjukova J. Holmqvist T. Danis A. S. Akerman K. E. Kukkonen J. P. (2004). Phospholipase C Activator m-3M3FBS Affects Ca2+ Homeostasis Independently of Phospholipase C Activation. Br. J. Pharmacol. 143 (1), 3–7. 10.1038/sj.bjp.0705911 15302681 PMC1575272

[B37] MacDougall M. R. Sharma S. (2020). Physiology, Chemoreceptor Trigger Zone. StatPearls. Treasure Island (FL). 30725818

[B38] Maeda S. Qu Q. Robertson M. J. Skiniotis G. Kobilka B. K. (2019). Structures of the M1 and M2 Muscarinic Acetylcholine receptor/G-Protein Complexes. Science 364 (6440), 552–557. 10.1126/science.aaw5188 31073061 PMC7034192

[B39] Michal P. El-Fakahany E. E. Doležal V. (2015). Changes in Membrane Cholesterol Differentially Influence Preferential and Non-preferential Signaling of the M1 and M3 Muscarinic Acetylcholine Receptors. Neurochem. Res. 40 (10), 2068–2077. 10.1007/s11064-014-1325-z 24821386 PMC4630253

[B40] Misra S. Murthy K. S. Zhou H. Grider J. R. (2004). Coexpression of Y1, Y2, and Y4 Receptors in Smooth Muscle Coupled to Distinct Signaling Pathways. J. Pharmacol. Exp. Ther. 311 (3), 1154–1162. 10.1124/jpet.104.071415 15308651

[B41] Murthy K. S. Makhlouf G. M. (1996). Opioid Mu, delta, and Kappa Receptor-Induced Activation of Phospholipase C-Beta 3 and Inhibition of Adenylyl Cyclase Is Mediated by Gi2 and G(o) in Smooth Muscle. Mol. Pharmacol. 50 (4), 870–877. 8863832

[B42] Navari R. M. (2013). Management of Chemotherapy-Induced Nausea and Vomiting : Focus on Newer Agents and New Uses for Older Agents. Drugs 73 (3), 249–262. 10.1007/s40265-013-0019-1 23404093

[B43] Navari R. M. (2014). Olanzapine for the Prevention and Treatment of Chronic Nausea and Chemotherapy-Induced Nausea and Vomiting. Eur. J. Pharmacol. 722, 180–186. 10.1016/j.ejphar.2013.08.048 24157985

[B44] Ng L. C. Wilson S. M. McAllister C. E. Hume J. R. (2007). Role of InsP3 and Ryanodine Receptors in the Activation of Capacitative Ca2+ Entry by Store Depletion or Hypoxia in Canine Pulmonary Arterial Smooth Muscle Cells. Br. J. Pharmacol. 152 (1), 101–111. 10.1038/sj.bjp.0707357 17592501 PMC1978272

[B45] Nie M. Selbie L. A. (1998). Neuropeptide Y Y1 and Y2 Receptor-Mediated Stimulation of Mitogen-Activated Protein Kinase Activity. Regul. Pept. 75-76, 207–213. 10.1016/s0167-0115(98)00070-6 9802411

[B46] Owusu Obeng E. Rusciano I. Marvi M. V. Fazio A. Ratti S. Follo M. Y. (2020) "Phosphoinositide-Dependent Signaling in Cancer: A Focus on Phospholipase C Isozymes." Int J Mol Sci 21(7). 10.3390/ijms21072581 PMC7177890 32276377

[B47] Parker S. L. Balasubramaniam A. (2008). Neuropeptide Y Y2 Receptor in Health and Disease. Br. J. Pharmacol. 153 (3), 420–431. 10.1038/sj.bjp.0707445 17828288 PMC2241788

[B48] Parsons M. E. Ganellin C. R. (2006). Histamine and its Receptors. Br. J. Pharmacol. 147 Suppl 1, S127–S135. 10.1038/sj.bjp.0706440 16402096 PMC1760721

[B49] Pedrosa R. Gomes P. Hopfer U. Jose P. A. Soares-da-Silva P. (2004). Gialpha3 Protein-Coupled Dopamine D3 Receptor-Mediated Inhibition of Renal NHE3 Activity in SHR Proximal Tubular Cells Is a PLC-PKC-Mediated Event. Am. J. Physiol. Ren. Physiol 287 (5), F1059–F1066. 10.1152/ajprenal.00139.2004 15265766

[B50] Ray A. P. Chebolu S. Darmani N. A. (2009). Receptor-selective Agonists Induce Emesis and Fos Expression in the Brain and Enteric Nervous System of the Least Shrew (Cryptotis Parva). Pharmacol. Biochem. Behav. 94 (1), 211–218. 10.1016/j.pbb.2009.08.010 19699757 PMC2771428

[B51] Rojas C. Raje M. Tsukamoto T. Slusher B. S. (2014). Molecular Mechanisms of 5-HT(3) and NK(1) Receptor Antagonists in Prevention of Emesis. Eur. J. Pharmacol. 722, 26–37. 10.1016/j.ejphar.2013.08.049 24184669

[B52] Rose P. M. Fernandes P. Lynch J. S. Frazier S. T. Fisher S. M. Kodukula K. (1995). Cloning and Functional Expression of a cDNA Encoding a Human Type 2 Neuropeptide Y Receptor. J. Biol. Chem. 270 (39), 22661–22664. 10.1074/jbc.270.39.22661 7559383

[B53] Rubovitch V. Gafni M. Sarne Y. (2003). The Mu Opioid Agonist DAMGO Stimulates cAMP Production in SK-N-SH Cells through a PLC-PKC-Ca++ Pathway. Brain Res. Mol. Brain Res. 110 (2), 261–266. 10.1016/s0169-328x(02)00656-3 12591162

[B54] Shen W. Martinez K. Chuang C. C. McIntosh M. (2013). The Phospholipase C Inhibitor U73122 Attenuates Trans-10, Cis-12 Conjugated Linoleic Acid-Mediated Inflammatory Signaling and Insulin Resistance in Human Adipocytes. J. Nutr. 143 (5), 584–590. 10.3945/jn.112.173161 23468551 PMC3738231

[B55] Smart D. Hirst R. A. Hirota K. Grandy D. K. Lambert D. G. (1997). The Effects of Recombinant Rat Mu-Opioid Receptor Activation in CHO Cells on Phospholipase C, [Ca2+]i and Adenylyl Cyclase. Br. J. Pharmacol. 120 (6), 1165–1171. 10.1038/sj.bjp.0701012 9134231 PMC1564574

[B56] Tan C. M. J. Green P. Tapoulal N. Lewandowski A. J. Leeson P. Herring N. (2018). The Role of Neuropeptide Y in Cardiovascular Health and Disease. Front. Physiol. 9, 1281. 10.3389/fphys.2018.01281 30283345 PMC6157311

[B57] Thore S. Dyachok O. Gylfe E. Tengholm A. (2005). Feedback Activation of Phospholipase C via Intracellular Mobilization and Store-Operated Influx of Ca2+ in Insulin-Secreting Beta-Cells. J. Cel Sci 118 (Pt 19), 4463–4471. 10.1242/jcs.02577 16159958

[B58] Triggle D. J. (2007). Calcium Channel Antagonists: Clinical Uses-Past, Present and Future. Biochem. Pharmacol. 74 (1), 1–9. 10.1016/j.bcp.2007.01.016 17276408

[B59] Wan C. P. Lau B. H. (1995). Neuropeptide Y Receptor Subtypes. Life Sci. 56 (13), 1055–1064. 10.1016/0024-3205(95)00041-4 9001438

[B60] Wickham R. J. (2020). Revisiting the Physiology of Nausea and Vomiting-Challenging the Paradigm. Support Care Cancer 28 (1), 13–21. 10.1007/s00520-019-05012-8 31388745

[B61] Zhao J. Deng Y. Jiang Z. Qing H. (2016). G Protein-Coupled Receptors (GPCRs) in Alzheimer's Disease: A Focus on BACE1 Related GPCRs. Front. Aging Neurosci. 8, 58. 10.3389/fnagi.2016.00058 27047374 PMC4805599

[B62] Zhong W. Chebolu S. Darmani N. A. (2021). Central and Peripheral Emetic Loci Contribute to Vomiting Evoked by the Akt Inhibitor MK-2206 in the Least Shrew Model of Emesis. Eur. J. Pharmacol. 900, 174065. 10.1016/j.ejphar.2021.174065 33775646 PMC8085164

[B63] Zhong W. Chebolu S. Darmani N. A. (2019). Intracellular Emetic Signaling Cascades by Which the Selective Neurokinin Type 1 Receptor (NK1R) Agonist GR73632 Evokes Vomiting in the Least Shrew (Cryptotis Parva). Neurochem. Int. 122, 106–119. 10.1016/j.neuint.2018.11.012 30453005 PMC6294657

[B64] Zhong W. Chebolu S. Darmani N. A. (2018). Intracellular Emetic Signaling Evoked by the L-type Ca2+ Channel Agonist FPL64176 in the Least Shrew (Cryptotis Parva). Eur. J. Pharmacol. 834, 157–168. 10.1016/j.ejphar.2018.06.035 29966616 PMC6104632

[B65] Zhong W. Chebolu S. Darmani N. A. (2016). Thapsigargin-induced Activation of Ca(2+)-CaMKII-ERK in Brainstem Contributes to Substance P Release and Induction of Emesis in the Least Shrew. Neuropharmacology 103, 195–210. 10.1016/j.neuropharm.2015.11.023 26631534

[B66] Zhong W. Hutchinson T. E. Chebolu S. Darmani N. A. (2014). Serotonin 5-HT3 Receptor-Mediated Vomiting Occurs via the Activation of Ca2+/CaMKII-dependent ERK1/2 Signaling in the Least Shrew (Cryptotis Parva). PLoS One 9 (8), e104718. 10.1371/journal.pone.0104718 25121483 PMC4133232

[B67] Zhong W. Picca A. J. Lee A. S. Darmani N. A. (2017). Ca2+ Signaling and Emesis: Recent Progress and New Perspectives. Auton. Neurosci. 202, 18–27. 10.1016/j.autneu.2016.07.006 27473611

[B68] Ziffert I. Kaiser A. Babilon S. Mörl K. Beck-Sickinger A. G. (2020). Unusually Persistent Gαi-Signaling of the Neuropeptide Y2 Receptor Depletes Cellular Gi/o Pools and Leads to a Gi-Refractory State. Cell Commun Signal 18 (1), 49. 10.1186/s12964-020-00537-6 32223755 PMC7104545

[B69] Zuccotti A. Clementi S. Reinbothe T. Torrente A. Vandael D. H. Pirone A. (2011). Structural and Functional Differences between L-type Calcium Channels: Crucial Issues for Future Selective Targeting. Trends Pharmacol. Sci. 32 (6), 366–375. 10.1016/j.tips.2011.02.012 21450352

