# Supplementary figures and images for: The Contribution of Phospholipase C in Vomiting in the Least Shrew (Cryptotis Parva) Model of Emesis
Source: Front Pharmacol. 2021 Sep 10;12:736842. doi: 10.3389/fphar.2021.736842 (PMC8461300; doi:10.3389/fphar.2021.736842)

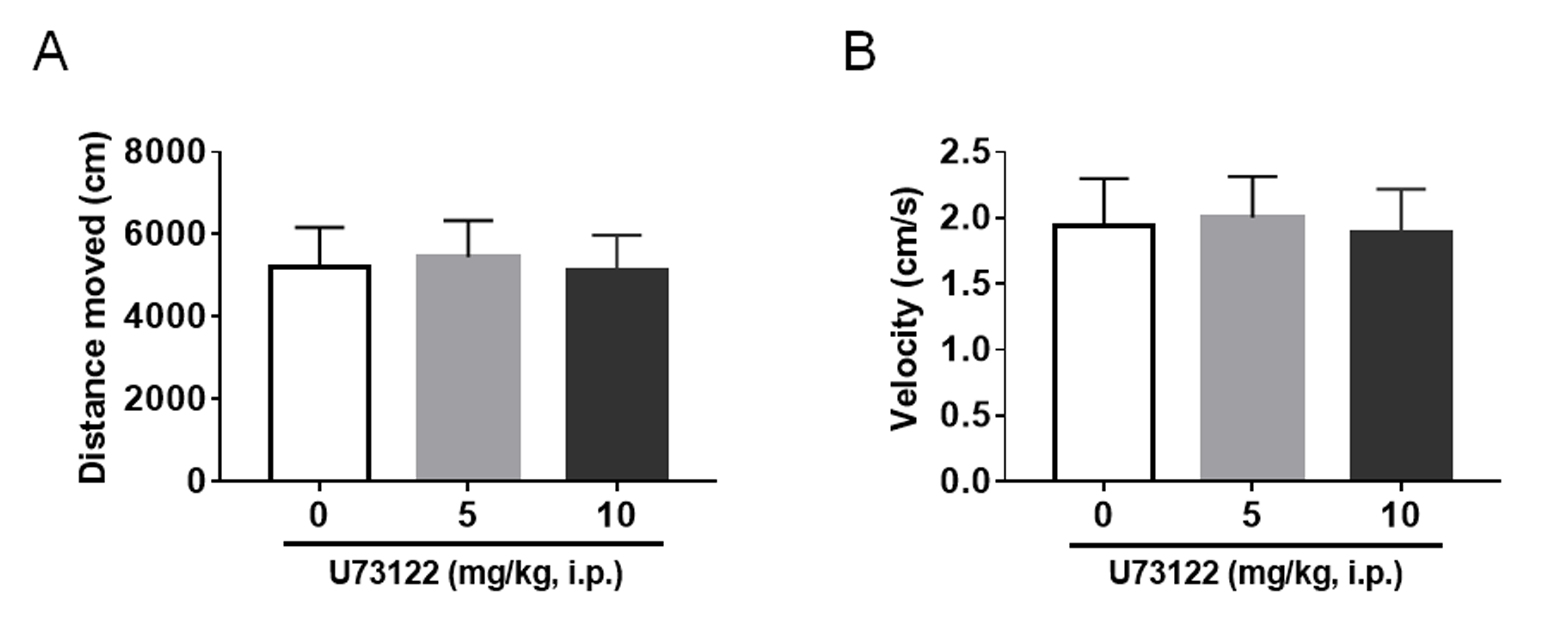

Supplement: Supplementary file 1 [file Image1.TIF]
